# Cardiovascular disease risk factor responses to a type 2 diabetes care model including nutritional ketosis induced by sustained carbohydrate restriction at 1 year: an open label, non-randomized, controlled study

**DOI:** 10.1186/s12933-018-0698-8

**Published:** 2018-05-01

**Authors:** Nasir H. Bhanpuri, Sarah J. Hallberg, Paul T. Williams, Amy L. McKenzie, Kevin D. Ballard, Wayne W. Campbell, James P. McCarter, Stephen D. Phinney, Jeff S. Volek

**Affiliations:** 1 Virta Health, San Francisco, CA USA; 2 Medically Supervised Weight Loss, Indiana University Health Arnett, Lafayette, IN USA; 3 Independent Consultant, Lafayette, CA USA; 4 Department of Kinesiology and Health, Miami University, Oxford, OH USA; 5 Department of Nutrition Science, Purdue University, West Lafayette, IN USA; 6 Department of Genetics, Washington University School of Medicine, St. Louis, MO USA; 7 Department of Human Sciences, The Ohio State University, Columbus, OH USA

**Keywords:** Ketosis, Carbohydrate restriction, Type diabetes, Cardiovascular disease, Risk factor, Atherogenic dyslipidemia, Inflammation, Blood pressure, Antihypertensive medication, Continuous remote care

## Abstract

**Background:**

Cardiovascular disease (CVD) is a leading cause of death among adults with type 2 diabetes mellitus (T2D). We recently reported that glycemic control in patients with T2D can be significantly improved through a continuous care intervention (CCI) including nutritional ketosis. The purpose of this study was to examine CVD risk factors in this cohort.

**Methods:**

We investigated CVD risk factors in patients with T2D who participated in a 1 year open label, non-randomized, controlled study. The CCI group (n = 262) received treatment from a health coach and medical provider. A usual care (UC) group (n = 87) was independently recruited to track customary T2D progression. Circulating biomarkers of cholesterol metabolism and inflammation, blood pressure (BP), carotid intima media thickness (cIMT), multi-factorial risk scores and medication use were examined. A significance level of P < 0.0019 ensured two-tailed significance at the 5% level when Bonferroni adjusted for multiple comparisons.

**Results:**

The CCI group consisted of 262 participants (baseline mean (SD): age 54 (8) year, BMI 40.4 (8.8) kg m^−2^). Intention-to-treat analysis (% change) revealed the following at 1-year: total LDL-particles (LDL-P) (− 4.9%, P = 0.02), small LDL-P (− 20.8%, P = 1.2 × 10^−12^), LDL-P size (+ 1.1%, P = 6.0 × 10^−10^), ApoB (− 1.6%, P = 0.37), ApoA1 (+ 9.8%, P < 10^−16^), ApoB/ApoA1 ratio (− 9.5%, P = 1.9 × 10^−7^), triglyceride/HDL-C ratio (− 29.1%, P < 10^−16^), large VLDL-P (− 38.9%, P = 4.2 × 10^−15^), and LDL-C (+ 9.9%, P = 4.9 × 10^−5^). Additional effects were reductions in blood pressure, high sensitivity C-reactive protein, and white blood cell count (all P < 1 × 10^−7^) while cIMT was unchanged. The 10-year atherosclerotic cardiovascular disease (ASCVD) risk score decreased − 11.9% (P = 4.9 × 10^−5^). Antihypertensive medication use was discontinued in 11.4% of CCI participants (P = 5.3 × 10^−5^). The UC group of 87 participants [baseline mean (SD): age 52 (10) year, BMI 36.7 (7.2) kg m^−2^] showed no significant changes. After adjusting for baseline differences when comparing CCI and UC groups, significant improvements for the CCI group included small LDL-P, ApoA1, triglyceride/HDL-C ratio, HDL-C, hsCRP, and LP-IR score in addition to other biomarkers that were previously reported. The CCI group showed a greater rise in LDL-C.

**Conclusions:**

A continuous care treatment including nutritional ketosis in patients with T2D improved most biomarkers of CVD risk after 1 year. The increase in LDL-cholesterol appeared limited to the large LDL subfraction. LDL particle size increased, total LDL-P and ApoB were unchanged, and inflammation and blood pressure decreased.

*Trial registration* Clinicaltrials.gov: NCT02519309. Registered 10 August 2015

**Electronic supplementary material:**

The online version of this article (10.1186/s12933-018-0698-8) contains supplementary material, which is available to authorized users.

## Background

Despite advances in the prevention and treatment of cardiovascular disease (CVD), it remains the leading cause of death in adults across the world [1]. Specifically, among those with type 2 diabetes (T2D) in the US, CVD accounts for 44% of mortality [2]. T2D rates have doubled over the past 20 years [3] and CVD risk increases two to fourfold with a diagnosis of T2D [4], warranting the identification of novel interventions to combat T2D. Intensive lifestyle interventions with dietary carbohydrate restriction [5–8], including the recently described continuous remote care model, which helps patients with T2D sustain nutritional ketosis [9, 10], have demonstrated improved glycemic control concurrent with medication reduction. However, the long-term sustainability and impact of these interventions on CVD risk and lipid profiles remains a subject of debate [11, 12].

Atherogenic dyslipidemia, a known risk factor for CVD [13], is highly prevalent in patients with T2D [14] and tightly linked to high-carbohydrate diets [15]. The condition is characterized by increased triglycerides, decreased high-density lipoprotein cholesterol concentration (HDL-C) and increased small low-density lipoprotein particle number (small LDL-P). Evidence suggests that increased very low-density lipoprotein particle number (VLDL-P), and in particular large VLDL-P, may be one of the key underlying abnormalities in atherogenic dyslipidemia [14, 16–18]. Elevated concentrations of small LDL are often associated with increased total LDL particle number (LDL-P) and ApoB [19, 20]. Particularly in patients with insulin resistance and T2D, elevated LDL-P and ApoB may exist even with normal to low LDL-C values [19, 21, 22]. Reliance on LDL-C for risk assessment in T2D patients may miss the impact of atherogenic dyslipidemia and elevated LDL-P. Researchers have proposed that LDL-P or ApoB may be superior to LDL-C as a predictor of CVD [22–25].

Previous studies of carbohydrate restriction of up to 1-year found a consistent decrease in triglycerides and increase in HDL-C, while LDL-C slightly increased or decreased [15, 26–28]. Although LDL-C is a risk factor for CVD, low LDL-C may belie elevations in small LDL, LDL-P or ApoB. Conversely, increased LDL-C with a low carbohydrate diet may primarily reflect the large LDL subfraction and may not increase CVD risk if total LDL-P or ApoB concentrations are unchanged or decline.

Inflammation, as assessed by elevated high-sensitivity C-reactive protein (hsCRP) or white blood cell count (WBC) [29–32], is an independent CVD risk factor and is involved in all stages of atherogenesis [33]. Inflammation is often observed in T2D concurrent with atherogenic dyslipidemia [34] and represents an additional CVD risk even in individuals with low to normal LDL-C [35, 36]. Hypertension is an additive risk factor in this patient population. Tighter blood pressure control has been associated with reduction in the risk of deaths related to diabetes. This included decreased CVD, stroke and microvascular complications [37].

For this open label, non-randomized, controlled, before-and-after study, we investigated the effects of a continuous care intervention (CCI) on CVD risk factors. The CCI included individualized digital support with telemedicine, health coaching, education in nutritional ketosis, biometric feedback, and an online peer-support community. Given the multi-faceted pathophysiology of CVD, we assessed the 1-year responses in several biomarkers related to cholesterol and lipoprotein metabolism, blood pressure, and inflammation, as well as carotid intima media thickness (cIMT) and medication use. Some results were previously reported in relation to glycemic control [10] and are presented here as they pertain to the effectiveness of the intervention and CVD risk (i.e. body weight and hemoglobin A1c).

## Methods

### Intervention

As previously described [9, 10], we utilized a prospective, longitudinal study design with a cohort of patients with T2D from the greater Lafayette, Indiana, USA, region who self-selected to participate in the CCI (Clinicaltrials.gov Identifier NCT02519309). Participants in the CCI were provided access to a web-based software application (app) for biomarker reporting and monitoring including body weight, blood glucose and blood betahydroxybutyrate (BHB; a marker of ketosis). The remote care team consisted of a health coach and physician or nurse practitioner who provided nutritional advice and medication management, respectively. Participants were guided by individualized nutrition recommendations to achieve and sustain nutritional ketosis. Notably, if participants reported headaches, constipation or lightheadedness, the remote care team recommended individualized adjustments to sodium and fluid intake [10]. CCI participants self-selected to receive education via either an onsite group setting (CCI-onsite) or via the app (CCI-web). There were no instructions given to the CCI group on counting or restricting calories. The CCI participants were instructed to restrict carbohydrate, eat protein in moderation, and consume fat to satiety from the start of the study. Due to the well-known systematic errors associated with dietary records in an obese population [38], we chose not to collect diet records. Social support was provided via an online peer community. Inclusion and exclusion criteria were previously described [10]. This study was approved by the Franciscan Health Lafayette Institutional Review Board, and participants provided written informed consent.

The frequency of glucose and BHB monitoring, along with glycemic control medication management, were previously described in detail [9, 10]. Briefly, glucose and BHB levels were initially obtained daily using a blood glucose and ketone meter (Precision Xtra, Abbott; Alameda, CA, USA) to personalize nutrition recommendations and also provide a marker of adherence. The frequency of measurement was modified by the care team for each participant based on individual care needs and preferences. For participants with a history of hypertension, a home automatic sphygmomanometer was supplied. Participants reported their home readings in the app daily to weekly depending on recent control and instruction from the supervising physician. Antihypertensive prescriptions were adjusted based on home readings and reported symptoms. Health coaches responded to patient app reported readings of systolic blood pressure less than 110 mmHg with specific questions about symptoms of hypotension. Following resolution of hypertension, diuretics were the first antihypertensive medications to be discontinued, followed by beta blockers, unless the participant had a history of coronary artery disease. Angiotensin-converting-enzyme inhibitors (ACEs) and angiotensin II receptor blockers (ARBs) were generally continued due to known renal protection with diabetes [39, 40]. Statin medications were adjusted when appropriate to maintain a goal of LDL-P under 1000 nmol L^−1^ or participant preference after full risk—benefit discussion.

To track T2D progression in the same geography and health system as the CCI, an independent cohort of patients with T2D who received usual care (UC) were recruited. These patients were referred to registered dietitians providing dietary advice according to American Diabetes Association guidelines [41].

### Outcome measures

Anthropometrics and vital signs for the CCI group were obtained at baseline, 70 days, and 1 year. A stadiometer was used to assess height and used in the calculation of body mass index. A calibrated scale in the clinic measured weight to the nearest 0.1 lb (Model 750, Detecto; Webb City, MO, USA) and values were converted to kg. Participants were provided a cellular-connected home scale for daily weight. Blood pressure was obtained manually by trained staff after participants rested in a seated position for 5 min. Adverse events were reported and reviewed by the Principal Investigator and the Institutional Review Board.

Fasting blood draws for the CCI group were collected at baseline, 70 days, and 1-year follow-up (ranging from 11 to 15 months). Blood analytes were determined via standard procedures at a Clinical Laboratory Improvement Amendment (CLIA) accredited laboratory on the day of sample collection or from stored serum. Serum aliquots were stored at − 80 °C and thawed for determination of ApoB, ApoA1, total cholesterol, triglycerides, and direct HDL-C concentrations by FDA approved methods (Cobas c501, Roche Diagnostics; Indianapolis, IN, USA). LDL was calculated using the Friedewald equation [42]. Lipid subfractions were quantified using clinical NMR LipoProfile^®^ (LabCorp, Burlington NC, USA; [43]). The LipoProfile3 algorithm used in the present investigation was used previously to relate lipid subfractions to CVD risk [35, 44, 45]. The NMR-derived lipoprotein insulin resistance score (LP-IR) is proposed to be associated with the homeostasis model assessment of insulin resistance (HOMA-IR) and glucose disposal rate (GDR) [46]. The multifactorial 10-year atherosclerotic cardiovascular disease (ASCVD) risk score was also computed [47].

Anthropometrics, vital signs and fasting blood draws for the UC group were obtained at baseline and 1 year as described above using the same clinical facilities and laboratory and data collection methods. Home biometrics for the UC group were not tracked and 70-day outcomes were not measured.

Carotid ultrasonography for cIMT measure was performed at baseline and 1 year in CCI and UC groups to characterize atherosclerotic risk. Ultrasound technicians were trained according to protocols that were previously tested and used to assess subclinical atherosclerosis [48, 49]. The right and left common carotid arteries were imaged 1 cm distal to the carotid bulb using a L12-3 multi-frequency linear-array transducer attached to a high-resolution ultrasound system (Phillips EPIQ 5, Amsterdam, Netherlands). Longitudinal images were captured in three imaging planes: anterior, lateral, and posterior. Digital images were analyzed using edge-detection software (Carotid Analyzer for Research; Medical Imaging Application, Coralville, IA) to trace the lumen-intima and intima-medial boundaries of the artery. Analyses were performed by the same blinded investigator to obtain right and left mean arterial diameter and mean cIMT. The current study was powered to detect a ∆cIMT difference of 0.019 mm between the CCI and UC groups at alpha = 0.05 and power = 80%.

### Statistics

JMP software (version 5.1, SAS Institute; Cary, SC, USA) was used for all statistical analyses except multiple imputation. Multiple imputation using multivariate normal distribution, conducted with Stata software (version 11, StataCorp; College Station, TX, USA), was used to estimate means and standard errors describing the variability between imputations. Seven hundred imputations from multivariate normal regression were run to estimate the missing values (4% missing at baseline and 22% missing at 1 year). Two-sample t tests were used to test for significance of the differences in baseline biomarker values between groups. Two-sample t tests were also used to test for differences between 1-year changes between groups. Paired t tests and analysis of covariance (ANCOVA) when adjusted for baseline covariates (sex, age, baseline BMI, insulin use (user vs. non-user), and African–American race) were used to test for significance of within-group changes. A secondary analysis was conducted with the addition of smoking status as a sixth covariate. To reduce skewness before testing for significance, triglyceride, triglyceride/HDL-C ratio and hsCRP were first log-transformed, however aside from P values, the tables present results from the untransformed data. Percent change in a given biomarker was calculated as the mean difference value divided by the mean baseline value. The standard error of percent change of a given biomarker was calculated as the standard error of the change divided by the mean baseline value. Significant changes in proportions (e.g. medication use) were tested using McNemar’s test with continuity correction in completers, and linear regression of the changes in the dichotomous states when missing outcome data were imputed.

Throughout the manuscript, standard deviations are presented within parentheses and standard errors are presented following “±” symbol. Nominal significance levels (P) are presented in the tables; however, a significance level of P < 0.0019 ensures simultaneous significance at P < 0.05 for a Bonferroni adjustment for the 26 variables examined. Unless otherwise noted, results presented are intention-to-treat analyses (all starters) with missing values estimated by imputation. Some results are designated as completer analyses (excluding participants who withdrew or lacked biomarkers at 1 year).

## Results

### Baseline characteristics of participants

The baseline characteristics of the 262 T2D participants who began the CCI are shown in Table 1. There were no significant differences in baseline characteristics between groups self-selecting web-based (CCI-web) and onsite education (CCI-onsite) (Additional file 1: Table S1) nor were there significant differences in biomarker changes at 1 year between the groups (Additional file 2: Table S2). Therefore, results for the two groups were combined for further analyses.

**Table 1:** Baseline characteristics for participants in the continuous care intervention (CCI) and usual care (UC) groups

	All		Completers with data	
	N	Mean (SD) or ± SE	N	Mean (SD) or ± SE
Age (years)				
CCI-all education^a^	262	54 (8)	218	54 (8)
Usual care^a^	87	52 (10)	78	52 (10)
CCI-all vs. usual care^b^		1 ± 1		2 ± 1*
Female (%)				
CCI-all education^a^	262	66.8 ± 2.9	218	65.1 ± 3.2
Usual care^a^	87	58.6 ± 5.3	78	60.3 ± 5.5
CCI-all vs. usual care^b^		8.2 ± 6.0		4.9 ± 6.4
Smokers (%)				
CCI-all education^a^	211	3.8 ± 1.3	176	4.0 ± 1.5
Usual care^a^	87	14.9 ± 3.8	78	14.1 ± 3.9
CCI-all vs. usual care^b^		− 11.2 ± 4.0^†^		− 10.1 ± 4.2*
Weight-clinic (kg)				
CCI-all education^a^	257	116.5 (25.9)	184	115.4 (24.6)
Usual care^a^	83	105.6 (22.2)	69	106.8 (22.2)
CCI-all vs. usual care^b^		10.9 ± 2.9^‡^		8.6 ± 3.2^†^
BMI (kg m^−2^)				
CCI-all education^a^	257	40.4 (8.8)	184	39.9 (7.9)
Usual care^a^	83	36.7 (7.3)	69	37.1 (7.6)
CCI-all vs. usual care^b^		3.7 ± 1.0^‡^		2.7 ± 1.1^†^
Hemoglobin A1c (%)				
CCI-all education^a^	262	7.60 (1.50)	204	7.49 (1.40)
Usual care^a^	87	7.64 (1.76)	72	7.74 (1.82)
CCI-all vs. usual care^b^		−0.04 ± 0.21		−0.25 ± 0.24
Systolic blood pressure (mmHg)				
CCI-all education^a^	260	132 (14)	187	133 (15)
Usual care^a^	79	130 (14)	67	129 (13)
CCI-all vs. usual care^b^		2 ± 2		4 ± 2*
Diastolic blood pressure (mmHg)				
CCI-all education^a^	260	82 (8)	187	82 (8)
Usual care^a^	79	82 (9)	67	81 (8)
CCI-all vs. usual care^b^		0 ± 1		0 ± 1
ApoB (mg dL^−1^)				
CCI-all education^a^	248	105 (29)	186	103 (28)
Usual care^a^	79	107 (28)	59	106 (30)
CCI-all vs. usual care^b^		−2 ± 4		−2
ApoA1 (mg dL^−1^)				
CCI-all education^a^	248	146 (28)	185	146 (29)
Usual care^a^	79	149 (22)	59	148 (21)
CCI-all vs. usual care^b^		−3 ± 3		−2 ± 3
ApoB/ApoA1 ratio				
CCI-all education^a^	248	0.74 (0.23)	185	0.73 (0.23)
Usual care^a^	79	0.73 (0.23)	59	0.73 (0.25)
CCI-all vs. usual care^b^		0.01 ± 0.03		0.00 ± 0.04
Triglycerides (mg dL^−1^)				
CCI-all education^a^	247	197 (143)	186	201 (153)
Usual care^a^	79	283 (401)	59	297 (458)
CCI-all vs. usual care^b^		−86 ± 46*		−97 ± 61
LDL-C (mg dL^−1^)				
CCI-all education^a^	232	103 (33)	172	100 (33)
Usual care^a^	70	102 (36)	48	100 (38)
CCI-all vs. usual care^b^		1 ± 5		0 ± 6
HDL-C (mg dL^−1^)				
CCI-all education^a^	247	42 (13)	186	42 (14)
Usual care^a^	79	38 (11)	59	37 (11)
CCI-all vs. usual care^b^		5 ± 2^†^		5 ± 2^†^
Triglycerides/HDL-C ratio				
CCI-all education^a^	247	5.9 (7.1)	186	6.1 (7.9)
Usual care^a^	79	10.5 (23.2)	59	11.5 (26.5)
CCI-all vs. usual care^b^		−4.6 ± 2.6		−5.4 ± 3.5
Large VLDL-P (nmol L^−1^)				
CCI-all education^a^	259	10 (8)	203	9 (8)
Usual care^a^	83	12 (12)	68	12 (13)
CCI-all vs. usual care^b^		−2 ± 1		−2 ± 2
Total LDL-P (nmol L^−1^)				
CCI-all education^a^	259	1300 (465)	203	1296 (476)
Usual care^a^	83	1289 (511)	68	1243 (484)
CCI-all vs. usual care^b^		11 ± 63		52 ± 68
Small LDL-P (nmol L^−1^)				
CCI-all education^a^	259	774 (377)	203	778 (378)
Usual care^a^	83	719 (322)	68	699 (326)
CCI-all vs. usual care^b^		55 ± 42		789 ± 48
LDL-particle size (nm)				
CCI-all education^a^	259	20.30 (0.55)	201	20.3 (0.55)
Usual care^a^	83	20.33 (0.56)	68	20.32 (0.55)
CCI-all vs. usual care^b^		−0.03 ± 0.07		−0.03 ± 0.08
Total HDL-P (μmol L^−1^)				
CCI-all education^a^	259	31.3 (6.4)	203	31.7 (6.4)
Usual care^a^	83	29.9 (5.8)	68	30.2 (6.0)
CCI-all vs. usual care^b^		1.4 ± 0.8		1.5 ± 0.9
Large HDL-P (μmol L^−1^)				
CCI-all education^a^	259	4.3 (2.5)	203	4.2 (2.5)
Usual care^a^	83	3.8 (2.1)	68	3.8 (2.1)
CCI-all vs. usual care^b^		0.4 ± 0.3		0.4 ± 0.3
LP-IR score				
CCI-all education^a^	259	72 (17)	203	72 (18)
Usual care^a^	83	75 (16)	68	74 (17)
CCI-all vs. usual care^b^		−3 ± 2		−2 ± 2
C-reactive protein (mg L^−1^)				
CCI-all education^a^	249	8.5 (14.5)	193	9.0 (16.1)
Usual care^a^	85	8.9 (8.6)	70	9.1 (9.0)
CCI-all vs. usual care^b^		−0.3 ± 1.3		−0.1 ± 1.6
WBC				
CCI-all education^a^	260	7.2 (1.9)	204	7.1 (1.8)
Usual care^a^	86	8.1 (2.4)	72	8.3 (2.4)
CCI-all vs. usual care^b^		−0.9 ± 0.3^†^		−1.2 ± 0.3^§^
10-year ASCVD risk (%)				
CCI-all education^a^	198	11.1 (9.1)	135	12.1 (9.3)
Usual care^a^	72	11.8 (10.8)	55	11.4 (10.8)
CCI-all vs. usual care^b^		−0.6 ± 1.4		0.8 ± 1.6
CIMT-average (mm)				
CCI-all education^a^	236	0.681 (0.108)	144	0.692 (0.113)
Usual care^a^	84	0.681 (0.116)	68	0.680 (0.111)
CCI-all vs. usual care^b^		−0.001 ± 0.014		0.013 ± 0.016
Statin (%)				
CCI-all education^a^	262	50.0 ± 3.1	218	51.8 ± 3.4
Usual care^a^	87	58.6 ± 5.3	73	54.8 ± 5.8
CCI-all vs. usual care^b^		−8.6 ± 6.1		−3.0 ± 6.7
Any antihypertensive medication (%)				
CCI-all education^a^	262	67.2 ± 2.9	218	68.4 ± 3.2
Usual care^a^	87	52.9 ± 5.4	73	50.7 ± 5.9
CCI-all vs. usual care^b^		14.3 ± 6.1*		17.7 ± 6.7^†^
ACE or ARB (%)				
CCI-all education^a^	262	29.4 ± 2.8	218	28.0 ± 3.0
Usual care^a^	87	18.4 ± 4.2	73	16.4 ± 4.3
CCI-all vs. usual care^b^		11.0 ± 5.0*		11.5 ± 5.3*
Diuretics (%)				
CCI-all education^a^	262	40.8 ± 3.0	218	41.3 ± 3.3
Usual care^a^	87	29.9 ± 4.9	73	24.7 ± 5.0
CCI-all vs. usual care^b^		11.0 ± 5.8		16.6 ± 6.1^†^

Significant baseline difference between means or percentages are designated by the following symbols: * 0.05 > P ≥ 0.01, ^†^0.01 > P ≥ 0.001, ^‡^0.001 > P ≥ 0.0001, ^§^P < 0.0001

^a^ Mean and standard deviations for continuous variables, percents and standard errors for categorical variables

^b^ Difference between means or percentages ± 1 standard error of the difference

The baseline characteristics of participants with measurements at both baseline and 1 year were not significantly different from dropouts and participants with missing data after correcting for multiple comparisons (Additional file 1: Table S1). This suggests that multiple imputation may be appropriate for estimating missing values in order to estimate outcomes for all starters.

An independently recruited UC group of 87 T2D participants, which provided an observational comparison group from the same geography and health system, showed no significant differences from the CCI group in baseline characteristics except mean body weight and BMI were higher in the CCI versus the UC group (Table 1, P < 0.001).

### Changes in biomarkers of CVD risk at 1 year

**Fig. 1:**
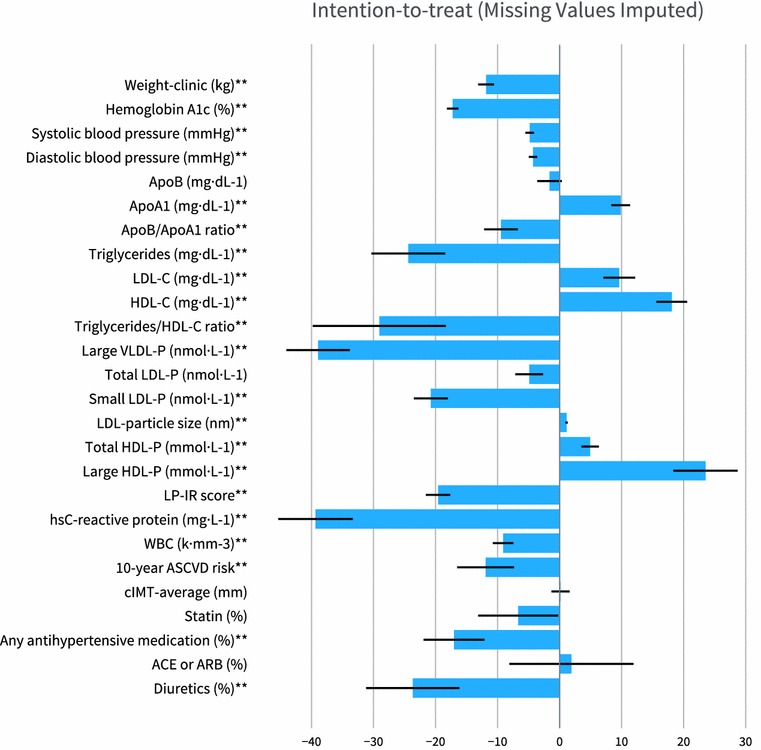
Change in biomarkers for CCI group. Bars indicate CCI group mean percent change in biomarkers based on the intention-to-treat analysis with missing values imputed. Percent change is computed as the change in mean values from baseline to 1 year divided by the mean baseline value. *Percent change* = 100 ×  [(*1* *year value* − *baseline value*)/(*baseline value*)]. Negative values indicate a decrease from baseline to 1 year while positive values indicate an increase. The ** symbol after the biomarker label indicates a statistically significant within group change from baseline (P < 0.0019, P adjusted for multiple comparisons). Error bars represent ± SE. *SE as Percent *= 100 × [(*1* *year value SE*)/(*baseline value*)]

Two-hundred eighteen (83%) participants remained enrolled in the CCI group at 1 year. One-year changes in CVD biomarkers are detailed in Table 2 and percent changes from baseline are shown in Fig. 1. The within-CCI group changes in the following lipids and lipoproteins were all statistically significant after adjusting for multiple comparisons (P < 0.0019), reported here as mean percent difference from baseline: ApoA1 (+ 9.8%), ApoB/ApoA1 ratio (− 9.5%), triglycerides (− 24.4%), LDL-C (+ 9.9%), HDL-C (+ 18.1%), triglyceride/HDL-C ratio (− 29.1%), large VLDL-P (− 38.9%), small LDL-P (− 20.8%), LDL-particle size (+ 1.1%), total HDL-P (+ 4.9%), and large HDL-P (+ 23.5%). There were no significant changes after adjusting for multiple comparisons in total LDL-P (− 4.9%, P = 0.02) or ApoB (− 1.6%, P = 0.37).

**Table 2:** 1-year biomarker changes for participants in the continuous care intervention group compared to usual care group

	Completers						All starters (dropouts imputed)^d^		
	N	1 year Mean ± SE	Unadjusted		Adjusted for baseline^c^		Unadjusted		
			Difference (SD) or ± SE	Significance^e^	Difference ± SE	Significance^e^	1 year Mean ± SE	Difference ± SE	Significance^e^
∆Weight-clinic (kg)									
CCI-all education^a^	184	101.2 ± 1.6	− 14.2 (10.3)	< 10^−16^	− 13.8 ± 0.6	< 10^−16^	102.7 ± 1.5	− 13.8 ± 0.7	< 10^−16^
Usual care^a^	69	106.8 ± 2.7	0.04 (5.9)	0.95	− 1.1 ± 1.1	0.29	107.3 ± 2.6	− 0.2 ± 0.8	0.85
CCI-all vs. usual care^b^			− 14.3 ± 1.0	< 10^−16^	− 12.7 ± 1.3	< 10^−16^		− 13.7 ± 1.1	< 10^−16^
∆Hemoglobin A1c (%)									
CCI-all education^a^	204	6.20 ± 0.07	− 1.29 (1.32)	< 10^−16^	− 1.32 ± 0.09	< 10^−16^	6.29 ± 0.07	− 1.30 ± 0.09	< 10^−16^
Usual care^a^	72	7.94 ± 0.22	0.20 (1.35)	0.21	0.22 ± 0.16	0.17	7.84 ± 0.19	0.20 ± 0.15	0.18
CCI-all vs. usual care^b^			− 1.49 ± 0.18	4.4 × 10^−16^	− 1.54 ± 0.19	4.4 × 10^−16^		− 1.50 ± 0.17	< 10^−16^
∆Systolic blood pressure (mmHg)									
CCI-all education^a^	187	126 ± 1	− 7 (16)	1.3 × 10^−8^	− 7 ± 1	1.6 × 10^−7^	126 ± 1	− 6 ± 1	1.3 × 10^−8^
Usual care^a^	67	129 ± 2	0 (18)	0.91	0 ± 2	0.83	129 ± 2	− 1 ± 2	0.67
CCI-all vs. usual care^b^			− 7 ± 2	0.005	− 6 ± 3	0.02		− 5 ± 2	0.02
∆Diastolic blood pressure (mmHg)									
CCI-all education^a^	187	78 ± 1	− 4 (9)	1.4 × 10^−7^	− 4 ± 1	6.2 × 10^−7^	79 ± 1	− 4 ± 1	7.2 × 10^−8^
Usual care^a^	67	81 ± 1	0 (10)	0.92	0 ± 1	0.75	81 ± 1	− 1 ± 1	0.45
CCI-all vs. usual care^b^			− 3 ± 1	0.01	− 3 ± 1	0.03		− 3 ± 1	0.06
∆ApoB (mg dL^−1^)									
CCI-all education^a^	186	103 ± 2	− 1 (24)	0.69	− 0 ± 2	0.82	104 ± 2	− 2 ± 2	0.37
Usual care^a^	59	107 ± 5	2 (37)	0.75	1 ± 4	0.9	106 ± 4	0 ± 4	0.95
CCI-all vs. usual care^b^			− 2 ± 5	0.66	− 1 ± 5	0.83		− 2 ± 5	0.67
∆ApoA1 (mg dL^−1^)									
CCI-all education^a^	185	160 ± 3	14 (24)	8.9 × 10^−16^	14 ± 2	4.4 × 10^−16^	160 ± 2	14 ± 2	< 10^−16^
Usual care^a^	59	145 ± 3	− 3 (19)	0.18	− 2 ± 3	0.55	147 ± 3	− 2 ± 3	0.37
CCI-all vs. usual care^b^			18 ± 3	4.7 × 10^−9^	16 ± 4	2.2 × 10^−5^		17 ± 3	1.4 × 10^−7^
∆ApoB/ApoA1									
CCI-all education^a^	185	0.67 ± 0.02	− 0.06 (0.17)	1.8 × 10^−6^	− 0.06 ± 0.02	0.003	0.67 ± 0.02	− 0.07 ± 0.01	1.9 × 10^−7^
Usual care^a^	59	0.76 ± 0.04	0.03 (0.29)	0.42	0.02 ± 0.03	0.5	0.74 ± 0.03	0.02 ± 0.03	0.58
CCI-all vs. usual care^b^			− 0.09 ± 0.04	0.02	− 0.08 ± 0.03	0.02		− 0.09 ± 0.03	0.01
∆Triglycerides (mg dL^−1^)									
CCI-all education^a^	186	151 ± 11	− 49 (168)	5.6 × 10^−5^	− 50 ± 16	0.001	148 ± 12	− 48 ± 13	< 10^−16^
Usual care^a^	59	327 ± 65	30 (301)	0.44	31 ± 29	0.27	305 ± 48	28 ± 32	0.43
CCI-all vs. usual care^b^			− 80 ± 41	0.05	− 81 ± 33	0.02		− 76 ± 35	9.9 × 10^−7^
∆LDL-C (mg dL^−1^)									
CCI-all education^a^	172	111 ± 3	11 (32)	7.7 × 10^−6^	11 ± 3	2.6 × 10^−5^	113 ± 3	10 ± 2	4.9 × 10^−5^
Usual care^a^	48	90 ± 4	− 11 (38)	0.05	− 11 ± 5	0.03	90 ± 5	− 11 ± 5	0.02
CCI-all vs. usual care^b^			22 ± 6	0.0003	22 ± 6	0.0002		21 ± 5	9.9 × 10^−5^
∆HDL-C (mg dL^−1^)									
CCI-all education^a^	186	50 ± 1	8 (12)	< 10^−16^	7 ± 1	< 10^−16^	50 ± 1	8 ± 1	< 10^−16^
Usual care^a^	59	35 ± 2	− 2 (9)	0.15	− 1 ± 2	0.69	37 ± 2	− 1 ± 1	0.41
CCI-all vs. usual care^b^			9 ± 1	1.7 × 10^−10^	8 ± 2	9.9 × 10^−6^		9 ± 2	1.2 × 10^−8^
Triglycerides/HDL-C ratio									
CCI-all education^a^	186	4.3 ± 0.6	− 1.8 (9.4)	< 10^−16^	− 1.9 ± 0.9	< 10^−16^	4.1 ± 0.6	− 1.6 ± 0.7	< 10^−16^
Usual care^a^	59	12.5 ± 2.7	0.9 (16.1)	0.1	1.2 ± 1.6	0.16	11.2 ± 2.1	1.0 ± 1.7	0.24
CCI-all vs. usual care^b^			− 2.8 ± 2.2	3.1 × 10^−10^	− 3.1 ± 1.8	5.5 × 10^−7^		− 2.6 ± 1.8	4.5 × 10^−9^
∆Large VLDL-P (nmol L^−1^)									
CCI-all education^A^	203	6 ± 1	− 4 (7)	5.6 × 10^−15^	− 4 ± 1	1.6 × 10^−14^	6 ± 1	− 4 ± 1	4.2 × 10^−15^
Usual care^a^	68	12 ± 2	0 (8)	0.71	0 ± 1	0.60	12 ± 1	0 ± 1	0.77
CCI-all vs. usual care^b^			− 3 ± 1	0.001	− 3 ± 1	0.002		3 ± 1	0.0007
∆Total LDL-P (nmol L^−1^)									
CCI-all education^a^	203	1234 ± 30	− 62 (375)	0.02	− 57 ± 29	0.05	1235 ± 29	− 64 ± 26	0.02
Usual care^a^	68	1196 ± 60	− 47 (491)	0.43	− 67 ± 53	0.21	1231 ± 57	− 57 ± 56	0.31
CCI-all vs. usual care^b^			− 15 ± 65	0.82	10 ± 62	0.87		− 7 ± 62	0.91
∆Small LDL-P (nmol L^−1^)									
CCI-all education^a^	203	614 ± 22	− 164 (332)	2.2 × 10^−12^	− 161 ± 24	4.1 × 10^−11^	613 ± 21	− 161 ± 23	1.2 × 10^−12^
Usual care^a^	68	724 ± 44	25 (370)	0.57	16 ± 45	0.71	740 ± 41	18 ± 42	0.67
CCI-all vs. usual care^b^			− 189 ± 51	0.0002	− 177 ± 52	0.0007		− 179 ± 48	0.0002
∆LDL-particle size (nm)									
CCI-all education^a^	201	20.53 ± 0.04	0.23 (0.54)	1.7 × 10^−9^	0.23 ± 0.04	8.9 × 10^−9^	20.53 ± 0.04	0.23 ± 0.04	6.0 × 10^−10^
Usual care^a^	68	20.25 ± 0.07	− 0.08 (0.53)	0.24	− 0.08 ± 0.07	0.25	20.25 ± 0.07	− 0.07 ± 0.06	0.25
CCI-all vs. usual care^b^			0.30 ± 0.07	4.4 × 10^−5^	0.31 ± 0.08	0.0002		0.30 ± 0.07	3.8 × 10^−15^
∆Total HDL-P (µmol L^−1^)									
CCI-all education^a^	203	33.2 ± 0.5	1.5 (4.9)	1.2 × 10^−5^	1.5 ± 0.4	2.1 × 10^−5^	32.8 ± 0.4	1.5 ± 0.3	5.6 × 10^−6^
Usual care^a^	68	29.4 ± 0.8	− 0.8 (4.7)	0.15	− 0.8 ± 0.6	0.23	29.2 ± 0.7	− 0.7 ± 0.6	0.23
CCI-all vs. usual care^b^			2.3 ± 0.7	0.0004	2.3 ± 0.7	0.003		2.2 ± 0.7	0.0008
∆Large HDL-P (µmol L^−1^)									
CCI-all education^a^	203	5.3 ± 0.2	1.0 (2.2)	2.5 × 10^−11^	1.0 ± 0.2	4.1 × 10^−11^	5.3 ± 0.2	1.0 ± 0.2	1.2 × 10^−11^
Usual care^a^	68	3.9 ± 0.3	0.1 (1.6)	0.69	0.2 ± 0.3	0.44	3.9 ± 0.3	0.1 ± 0.2	0.74
CCI-all vs. usual care^b^			0.9 ± 0.3	0.0002	0.8 ± 0.3	0.01		0.9 ± 0.3	0.0004
∆LP-IR score									
CCI-all education^a^	203	58 ± 2	− 14 (18)	< 10^−16^	− 14 ± 1	< 10^−16^	58 ± 1	− 14 ± 1	< 10^−16^
Usual care^a^	68	74 ± 2	− 1 (16)	0.73	− 2 ± 2	0.41	75 ± 2	− 1 ± 2	0.74
CCI-all vs. usual care^b^			− 13 ± 2	3.8 × 10^−9^	− 12 ± 3	6.2 × 10^−6^		− 13 ± 2	6.2 × 10^−9^
∆C-reactive protein (mg L^−1^)									
CCI-all education^a^	193	5.7 ± 0.5	− 3.3 (13.4)	< 10^−8^	− 3.1 ± 1.0	< 10^−16^	5.6 ± 0.6	− 3.6 ± 1.1	< 10^−16^
Usual care^a^	70	10.4 ± 1.8	1.3 (13.3)	0.94	0.9 ± 1.7	0.88	10.3 ± 1.6	1.3 ± 1.5	0.93
CCI-all vs. usual care^b^			− 4.7 ± 1.9	1.2 × 10^−6^	− 4.0 ± 2.0	3.0 × 10^−5^		− 4.9 ± 1.8	9.3 × 10^−7^
∆WBC (k mm^−3^)									
CCI-all education^a^	204	6.5 ± 0.1	− 0.7 (1.4)	2.1 × 10^−11^	− 0.7 ± 0.1	2.1 × 10^−11^	6.6 ± 0.1	− 0.7 ± 0.1	3.2 × 10^−11^
Usual care^a^	72	8.3 ± 0.3	− 0.1 (1.6)	0.76	− 0.1 ± 0.2	0.74	8.1 ± 0.3	− 0.1 ± 0.2	0.76
CCI-all vs. usual care^b^			− 0.6 ± 0.2	0.003	− 0.6 ± 0.2	0.004		− 0.6 ± 0.2	0.003
∆10-year ASCVD risk (%)									
CCI-all education^a^	135	10.5 ± 0.7	− 1.6 (5.4)	0.0004	− 1.5 ± 0.6	0.01	9.6 ± 0.5	− 1.3 ± 0.3	4.9 × 10^−5^
Usual care^a^	55	12.7 ± 1.5	1.4 (9.3)	0.28	1.1 ± 1.0	0.27	12.9 ± 1.2	1.2 ± 0.9	0.17
CCI-all vs. usual care^b^			− 3.0 ± 1.3	0.03	− 2.6 ± 1.2	0.03		− 2.6 ± 1.0	0.008
∆CIMT-average (mm)									
CCI-all education^a^	144	0.695 ± 0.009	0.002 (0.055)	0.63	0.003 ± 0.004	0.45	0.685 ± 0.010	0.002 ± 0.004	0.65
Usual care^a^	68	0.680 ± 0.013	0.004 (0.041)	0.37	0.002 ± 0.006	0.74	0.680 ± 0.013	0.001 ± 0.006	0.87
CCI-all vs. usual care^b^			− 0.002 ± 0.007	0.74	0.001 ± 0.008	0.87		0.001 ± 0.007	0.88
∆Statin (%)									
CCI-all education^a^	218	48.2 ± 3.4	− 3.7 (34.4)	0.12	− 3.6 ± 2.4	0.13	46.7 ± 3.2	− 3.3 ± 2.3	0.15
Usual care^a^	73	64.4 ± 5.6	9.6 (37.9)	0.03	9.5 ± 4.3	0.03	67.4 ± 5.4	8.8 ± 4.3	0.04
CCI-all vs. usual care^b^			− 13.3 ± 5.0	0.008	− 13.2 ± 5.0	0.009		− 12.1 ± 4.9	0.01
∆Any antihypertensive medication (%)									
CCI-all education^a^	218	56.4 ± 3.4	− 11.9 (42.3)	3.2 × 10^−5^	− 11.9 ± 2.9	3.6 × 10^−5^	55.8 ± 3.3	− 11.4 ± 2.8	5.3 × 10^−5^
Usual care^a^	73	60.3 ± 5.8	9.6 (41.4)	0.05	9.6 ± 5.1	0.06	61.2 ± 5.6	8.3 ± 4.8	0.09
CCI-all vs. usual care^b^			− 21.5 ± 5.6	0.0002	− 21.6 ± 6.0	0.0004		− 19.7 ± 5.6	0.0004
∆ACE or ARB (%)									
CCI-all education^a^	218	28.9 ± 3.1	0.9 (27.1)	0.62	1.5 ± 1.9	0.42	30.0 ± 2.9	0.6 ± 1.9	0.76
Usual care^a^	73	21.9 ± 4.9	5.5 (28.3)	0.1	3.7 ± 3.3	0.27	23.4 ± 4.7	5.0 ± 3.3	0.13
CCI-all vs. usual care^b^			− 4.6 ± 3.8	0.23	− 2.1 ± 3.9	0.59		− 4.4 ± 3.8	0.24
∆Diuretics (%)									
CCI-all education^a^	218	31.7 ± 3.2	− 9.6 (41.3)	0.0006	− 9.5 ± 2.7	0.0004	31.3 ± 3.1	− 9.7 ± 2.7	0.0004
Usual care^a^	73	30.1 ± 5.4	5.5 (32.9)	0.16	5.2 ± 4.8	0.28	33.0 ± 5.3	3.2 ± 4.1	0.44
CCI-all vs. usual care^b^			− 15.1 ± 4.8	0.001	− 14.7 ± 5.6	0.009		− 12.8 ± 4.9	0.009

^a^ Means (standard deviations) or ± one standard error are presented. Sample sizes, means, and significance levels refer to subjects with baseline and 1-year measurements for *completers*, and to 349 subjects (262 intervention and 87 usual care) for *all starters*. Significance levels for *completers* refer to one-sample t test with or without adjustment. Untransformed triglyceride and C-reactive protein values are presented, however, their statistical significances were based on their log-transformed values

^b^ Mean differences ± one standard error are presented. Significance levels refer to two-sample t test or analysis of covariance for the differences

^c^ Adjusted for sex, age, baseline BMI, baseline insulin use (user vs. non-user), and African–American race

^d^ Imputed values based on 700 iterations from multivariate normal regression

^e^ A significance level of P < 0.0019 ensures overall simultaneous significance of P <  0.05 over the 26 variables using Bonferroni correction

The CCI group experienced significant reductions in systolic BP (− 4.8%), diastolic BP (− 4.3%), hsCRP (− 39.3%) and WBC count (− 9.1%). Regarding medication changes, (reported here as percent use at 1 year minus percent use at baseline, while Fig. 1 displays percent change of percent use) significant reductions were observed in overall use of antihypertensive medication (− 11.4%) and diuretics (− 9.7%) whereas changes in ACE or ARB (0.6%) and statin (− 3.3%) use were not significant. Significant reductions were observed in both multivariate metrics: 10-year ASCVD risk (− 11.9%) and LP-IR (− 19.6%). There was no significant change in cIMT (averaged right and left values). In addition, changes in cIMT were not significantly correlated with baseline LDL-P or LDL-C, or changes in LDL-P or LDL-C (all P ≥ 0.33).

**Fig. 2:**
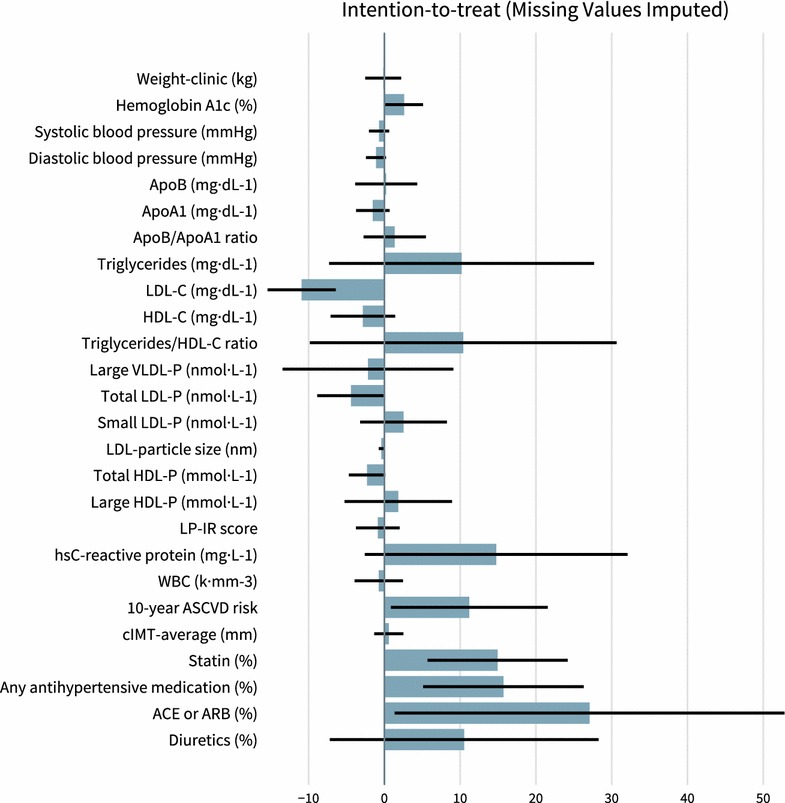
Change in biomarkers for UC group. Bars indicate UC group mean percent change in biomarkers based on the intention-to-treat analysis with missing values imputed. Percent change is computed as the change in mean values from baseline to 1 year divided by the mean baseline value. *Percent change* = 100 ×  [(*1* *year value* − *baseline value*)/(*baseline value*)]. Negative values indicate a decrease from baseline to 1 year while positive values indicate an increase. (None of the within group changes were statistically significant, i.e. all P > 0.0019, P adjusted for multiple comparisons.) Error bars represent ± SE. *SE as Percent *= 100 × [(*1* *year value SE*)/(*baseline value*)]

One-year results from the UC group are provided in Table 2 and Fig. 2. Within the UC group, after adjustment for multiple comparisons there were no significant changes at 1 year. After adjusting for differences in baseline characteristics (sex, age, baseline BMI, insulin use (user vs. non-user), and African–American race) and multiple comparisons, the changes observed at 1 year for the following biomarkers were significantly different between the CCI and UC groups (mean ∆CCI − mean ∆UC, where ∆ is 1 year minus baseline): small LDL-P (− 177 nmol L^−1^), ApoA1 (+ 16 mg dL^−1^), triglyceride/HDL-C ratio (− 3.1), LDL particle size (+ 0.31 nm), HDL-C (+ 8 mg dL^−1^), LDL-C (+ 22 mg dL^−1^), hsCRP (− 4.0 mg dL^−1^), and LP-IR (− 12). Adding smoking status to the list of covariates mentioned above did not lead to any changes in statistical significance.

There were no significant differences in change in biomarkers between the sexes within the CCI group or between CCI and UC groups among completers (all P > 0.0019). The results related to daily weight and ketone measurements were previously reported in detail [9, 10]. In brief, almost all CCI participants (96%) reported at least one BHB value ≥ 0.5 mmol L^−1^ by handheld measure. Laboratory-measured BHB at 1 year (0.31 ± 0.03 mmol L^−1^) was almost twice as large as the baseline average in the CCI group (0.17 ± 0.01 mmol L^−1^). For this population, additional details on changes in other biomarkers related to glycemic control, metabolic acidosis, and liver, kidney, and thyroid health were previously reported in greater detail [9, 10]. In addition, details on safety and adverse events have previously been described [10]. A post hoc analysis of covariance on treatment versus control group differences in 1-year risk factor change suggested that weight loss was associated with as much as approximately 40–70% of the change in the following biomarkers: small LDL-P, ApoA1, triglyceride/HDL-C ratio, triglycerides, and HDL-C and over 90% of the difference in LP-IR score.

### Range of outcomes

The distribution and range of intervention response for the CCI and UC groups were compared for LDL-P, small LDL-P, large VLDL-P, ApoB, ApoA1, ApoB/ApoA1 ratio, and TG/HDL-C ratio (Additional file 3: Figure S1). Ranges of change observed in the CCI group were within the ranges observed in the UC group for increases in LDL-P, small LDL-P, ApoB and ApoB/ApoA1 ratio. There were two CCI participants (2/203, 1.0%) whose change in large VLDL-P exceeded the maximum observed in the UC group (15.2 nmol L^−1^). There was one CCI participant (1/185, 0.5%) whose change in ApoA1 was less than the minimum observed in the UC group (− 58 mg dL^−1^) and one CCI participant (1/186, 0.5%) whose change in triglyceride/HDL-C ratio was higher than the maximum observed in the UC group (64.9).

## Discussion

This study demonstrates that a CCI utilizing remote physician and health coach support with nutritional ketosis beneficially altered most CVD risk factors in patients with T2D at 1 year. Changes included: decreased small LDL-P, triglycerides, blood pressure and antihypertensive medication, hsCRP, and WBC count; increased HDL-C and LDL particle size; no change in LDL-P, ApoB, and cIMT and an increase in LDL-C. Combined with the previously reported improvements in glycemic control and reduction in obesity [10], which reduce CVD risk [50], these results demonstrate multiple additional benefits of the CCI with the exception of increased LDL-C.

Studies of dietary carbohydrate restriction, with a presumed increase in saturated fat intake, have shown modest changes in LDL-C levels [15, 26–28, 51]. The mean 10 mg dL^−1^ change observed in the CCI group in this study is numerically higher than the upper range of values reported by meta-analysis of lipid changes over 1 year related to carbohydrate restriction (− 7 to + 7 mg dL^−1^) [52]. Higher LDL-C is related to increased CVD risk [53, 54], but also is *inversely* correlated with mortality in two large prospective studies and a systemic review [55–57]. Additionally, there is no evidence that increasing or decreasing LDL-C with diet interventions has any impact on mortality. LDL-C increased in the current study but both ApoB and LDL-P, measures found to be better predictors of CVD risk, did not change significantly [20–23, 25, 58]. In addition, the reduction in small LDL-P, increase in LDL size, and decrease in large VLDL-P that occurred in the present investigation are also associated with reduced CVD risk [59–61].

A decrease in triglycerides and increase in HDL-C has also been previously reported in studies of carbohydrate restriction [15, 26–28, 50]. In patients with elevated baseline triglycerides (≥ 200 mg dL^−1^), a decrease in triglycerides (− 21%) and increase in HDL-C (+ 18%), which is similar to the changes observed in the intervention group in this study, has been associated with decreased CVD events [62]. Taken together, the decrease in triglycerides and increase in LDL-C may be partly due to decreased cholesterol ester transfer protein (CETP) exchange. Further studies on underlying mechanisms will help elucidate the causal relationships between the various concurrent changes in lipoproteins.

While mean response of CCI participants demonstrated an improvement in most lipid biomarkers and CVD risk factors other than LDL-C, we investigated whether a minority of participants might have unfavorable responses to the intervention. Our results suggest that a small number of participants (≤ 1%) demonstrated changes at 1 year outside the range of what was observed in a usual care population (Additional file 3: Figure S1). Thus, these results counter the concern that a significant portion of participants may have an extremely adverse reaction to the CCI (due to presumed increase in saturated fat intake) as compared to UC.

Inflammation is directly involved in all aspects of the pathogenesis of CVD [33]. High-sensitivity CRP and WBC count are widely accepted markers of inflammation and risk factors for CVD [29–32]. In addition to reducing cholesterol, reduction in inflammation may be a secondary mechanism of statins in lowering CVD risk [63–65]. The present study demonstrated a 39% reduction of hsCRP and 9% reduction in WBC count in the CCI, indicating a significant reduction in inflammation at 1 year. This response may be due in part to suppression of the NLRP3 inflammasome by BHB [66].

The reduction of blood pressure with concurrent reduction in antihypertensive medication was also significant. Blood pressure goals were recently reduced [67] and strong evidence exists that elevated blood pressure is a primary cardiovascular risk factor [68]. An analysis of a large T2D population suggested that antihypertensive medication may have limited effectiveness in reducing the prevalence of hypertension in these patients [69], whereas a study of weight loss interventions showed that a decrease in blood pressure predicted regression of carotid vessel wall volume [70]. Thus, additional lifestyle interventions that can augment blood pressure reduction such as the CCI described here may reduce CVD events. Additionally, the antihypertensives that were primarily decreased in the current study were shown to increase the risk for diabetes [71]. Their removal may represent further metabolic benefit.

Carotid intima media thickness (cIMT) is a non-invasive measure of subclinical atherosclerosis that is significantly associated with CVD morbidity and mortality [48, 49, 72, 73]. However, a recent meta-analysis in 3902 patients with T2D found that cIMT progression over an average of 3.6 years did not correlate with CVD events [72]. We found no significant change in cIMT from baseline to 1 year in either the CCI or UC groups. Progression or regression of cIMT may take multiple years to manifest and may require a larger cohort to achieve statistical significance [73]. In summary, the cIMT results from this study provide no evidence of vascular harm or benefit from 1 year of nutritional ketosis in patients with T2D.

## Strengths and limitations of the study

Prior studies have demonstrated favorable improvements in atherogenic dyslipidemia with minimal or no change in LDL-C and LDL-P following managed ketogenic diets in small short-term randomized trials. This study’s strengths include its larger cohort with high retention, prospective design and 1-year duration. The study was the first to assess ApoB and ApoA1 in a T2D population adhering to a ketogenic diet. This study also has real-world application due to the outpatient setting without the use of meal replacements or food provisions.

Limitations of this study include the lack of randomization between the CCI and UC groups. In addition, the intervention provided to CCI participants was of greater intensity than UC. This was a single site study and the racial composition of study participants was predominantly Caucasian. The study was not of sufficient size and duration to determine significant differences in CVD morbidity or mortality. Since the intervention led to concurrent weight loss and improvements in cardiovascular health, it is difficult to conclude how much of the improvement can be attributed to weight loss versus other simultaneous physiological changes. In an attempt to assess the role of weight loss, a post hoc analysis of covariance on treatment versus control group differences in 1-year risk factor change suggested that weight loss was related to a large proportion of the change in: small LDL-P, ApoA1, triglyceride/HDL-C ratio, triglycerides, and HDL-C and LP-IR score. However, the results from a recent study comparing a low-fat diet group with a low-carbohydrate group, with similar weight loss at 12 months between groups, suggest that the role of weight loss may be more modest (the low-fat group showed only 15% of the HDL-C gain and 35% of the triglyceride decrease, relative to the low-carbohydrate group) [74]. Additional future studies that tightly control weight loss (and other possible mechanisms for reduction in CVD risk, e.g. diet, smoking, genetic factors, stress, etc.) would lead to better estimates of how much weight loss independently contributes to the improvements observed in the intervention group relative to other factors. Furthermore, future trials could include a longer multi-site, randomized controlled trial to allow for hard end point evaluation. Greater racial and ethnic diversity, a broader age range, and greater disease severity could also be evaluated.

## Conclusions

A T2D intervention combining technology-enabled continuous remote care with individualized plans encouraging nutritional ketosis has demonstrated diabetes status improvement while improving many CVD risk factors including atherogenic dyslipidemia, inflammation and blood pressure while decreasing use of antihypertensive mediations. Ongoing research will determine the continued safety, sustainability, and effectiveness of the intervention.

## Additional files

**Additional file 1: Table S1.** Detailed baseline characteristics for participants in the continuous care intervention (CCI) and usual care (UC) groups.

**Additional file 2: Table S2.** Details on 1-year biomarker changes for participants in the continuous care intervention group compared to usual care group.

**Additional file 3: Figure S1.** Distribution of changes in selected biomarkers for CCI and UC completers. Histograms of changes at one year for CCI (blue) and UC (gray) are overlaid. Very few (≤1%) CCI participants demonstrated changes in an undesirable direction at one year that were outside the range of changes observed in the UC group for key lipid and lipoprotein particles. (A) Apolipoprotein B (B) Apolipoprotein A1 (C) Apolipoprotein B/Apolipoprotein A1 ratio (D) LDL-P (E) Small LDL-P (F) Large VLDL-P (G) Triglyceride/HDL-C ratio.

## Abbreviations

ACE: angiotensin-converting-enzyme inhibitors
ApoA1: apolipoprotein A1
ApoB: apolipoprotein B
ARB: angiotensin II receptor blockers
ASCVD risk score: 10-year atherosclerotic cardiovascular disease risk score
BHB: beta-hydroxybutyrate
BP: blood pressure
CCI: continuous care intervention
CCI-onsite: subset of CCI participants who selected to receive onsite education
CCI-web: subset of CCI participants who selected to receive web-based education
cIMT: carotid intima media thickness
CVD: cardiovascular disease
GDR: glucose disposal rate
HOMA-IR: homeostatic model assessment of insulin resistance
hsCRP: high sensitive C-reactive protein
LP-IR: lipoprotein insulin resistance score
T2D: type 2 diabetes
UC: usual care
VLDL-P: very low-density lipoprotein particle number
WBC: white blood cell
