# Supplementary material for: Cardiovascular disease risk factor responses to a type 2 diabetes care model including nutritional ketosis induced by sustained carbohydrate restriction at 1 year: an open label, non-randomized, controlled study
Source: Cardiovasc Diabetol. 2018 May 1;17:56. doi: 10.1186/s12933-018-0698-8 (PMC5928595; doi:10.1186/s12933-018-0698-8)
Supplement: Supplementary file 1 — Additional file 1: Table S1. Detailed baseline characteristics for participants in the continuous care intervention (CCI) and usual care (UC) groups. [file 12933_2018_698_MOESM1_ESM.docx]

| Supplementary table 1. Detailed baseline characteristics for participants in the continuous care intervention (CCI) and usual care (UC) groups | | | | | | | |
| --- | --- | --- | --- | --- | --- | --- | --- |
|  | **All** | | **Completers with data** | | **Dropout or missing data** | | **Completers-Dropouts** |
|  | **N** | **Mean (SD) or ±SE** | **N** | **Mean (SD) or ±SE** | **N** | **Mean (SD) or ±SE** | **Mean±SE** |
| **Age (years)** |  |  |  |  |  |  |  |
| All | 349 | 53 (9) | 296 | 53 (9) | 53 | 53 (8) | 0 ± 1 |
| CCI-all education^1^ | 262 | 54 (8) | 218 | 54 (8) | 44 | 52 (8) | 2 ± 1 |
| CCI-web^1^ | 126 | 53 (8) | 104 | 53 (8) | 22 | 53 (8) | 1 ± 2 |
| CCI-onsite^1^ | 136 | 54 (9) | 114 | 55 (8) | 22 | 51 (8) | 3 ± 2 |
| Usual care^1^ | 87 | 52 (10) | 78 | 52 (10) | 9 | 58 (7) | -6 ± 3* |
| CCI-web vs. CCI-onsite^2^ |  | -1 ± 1 |  | -1 ± 1 |  | 2 ± 3 |  |
| CCI-web vs. usual care^2^ |  | 1 ± 1 |  | 2 ± 1 |  | -5 ± 3 |  |
| CCI-onsite vs. usual care^2^ |  | 12 ± 1 |  | 3 ± 1 |  | -7 ± 3 |  |
| CCI-all vs. usual care^2^ |  | 1 ± 1 |  | 2 ± 1* |  | -6 ± 3* |  |
|  |  |  |  |  |  |  |  |
| **Female (%)** |  |  |  |  |  |  |  |
| All | 349 | 64.8 ± 2.6 | 296 | 63.9 ± 2.8 | 53 | 69.8 ± 6.3 | -6.0 ± 6.9 |
| CCI-all education^1^ | 262 | 66.8 ± 2.9 | 218 | 65.1 ± 3.2 | 44 | 75.0 ± 6.5 | -9.9 ± 7.3 |
| CCI-web^1^ | 126 | 61.9 ± 4.3 | 104 | 57.7 ± 4.8 | 22 | 81.8 ± 8.2 | -24.1 ± 9.5* |
| CCI-onsite^1^ | 136 | 71.3 ± 3.9 | 114 | 71.9 ± 4.2 | 22 | 68.2 ± 9.9 | 3.8 ± 10.8 |
| Usual care^1^ | 87 | 58.6 ± 5.3 | 78 | 60.3 ± 5.5 | 9 | 44.4 ± 16.6 | 15.8 ± 17.5 |
| CCI-web vs. CCI-onsite^2^ |  | -9.4 ± 5.8 |  | -14.2 ± 6.4* |  | 13.6 ± 12.9 |  |
| CCI-web vs. usual care^2^ |  | 3.3 ± 6.8 |  | -2.6 ± 7.4 |  | 37.4 ± 18.5* |  |
| CCI-onsite vs. usual care^2^ |  | 12.7 ± 6.6* |  | 11.7 ± 7.0 |  | 23.7 ± 19.3 |  |
| CCI-all vs. usual care^2^ |  | 8.2 ± 6.0 |  | 4.9 ± 6.4 |  | 30.6 ± 17.8 |  |
|  |  |  |  |  |  |  |  |
| **Smokers (%)** |  |  |  |  |  |  |  |
| All | 298 | 7.1 ± 1.5 | 254 | 7.1 ± 1.6 | 44 | 6.8 ± 3.8 | 0.3 ± 4.1 |
| CCI-all education^1^ | 211 | 3.8 ± 1.3 | 176 | 4.0 ± 1.5 | 35 | 2.9 ± 2.8 | 1.1 ± 3.2 |
| CCI-web^1^ | 86 | 2.3 ± 1.6 | 71 | 2.8 ± 2.0 | 15 | 0.0 ± 0.0 | 2.8 ± 2.0 |
| CCI-onsite^1^ | 125 | 4.8 ± 1.9 | 105 | 4.8 ± 2.1 | 20 | 5.0 ± 4.9 | -0.2 ± 5.3 |
| Usual care^1^ | 87 | 14.9 ± 3.8 | 78 | 14.1 ± 3.9 | 9 | 22.2 ± 13.9 | -8.1 ± 14.4 |
| CCI-web vs. CCI-onsite^2^ |  | -2.5 ± 2.5 |  | -2.0 ± 2.9 |  | -5.0 ± 4.9 |  |
| CCI-web vs. usual care^2^ |  | -12.6 ± 4.2† |  | -11.3 ± 4.4† |  | -22.2 ± 13.9 |  |
| CCI-onsite vs. usual care^2^ |  | -10.1 ± 4.3* |  | -9.3 ± 4.5* |  | -17.2 ± 14.7 |  |
| CCI-all vs. usual care^2^ |  | -11.2 ± 4.0† |  | -10.1 ± 4.2* |  | -19.4 ± 14.1 |  |
|  |  |  |  |  |  |  |  |
| **Weight-clinic (kg)** |  |  |  |  |  |  |  |
| All | 340 | 113.9 (25.5) | 253 | 113.1 (24.2) | 87 | 116.1 (28.8) | -3.1 ± 3.4 |
| CCI-all education^1^ | 257 | 116.5 (25.9) | 184 | 115.4 (24.6) | 73 | 119.3 (29.0) | -3.8 ± 3.9 |
| CCI-web^1^ | 121 | 114.6 (27.1) | 84 | 114.8 (25.9) | 37 | 114.1 (30.0) | 0.6 ± 5.7 |
| CCI-onsite^1^ | 136 | 118.2 (24.9) | 100 | 116.0 (23.6) | 36 | 124.5 (27.4) | -8.5 ± 5.1 |
| Usual care^1^ | 83 | 105.6 (22.2) | 69 | 106.8 (22.2) | 14 | 99.9 (21.9) | 6.8 ± 6.4 |
| CCI-web vs. CCI-onsite^2^ |  | -3.6 ± 3.3 |  | -1.2 ± 3.7 |  | -10.4 ± 6.7 |  |
| CCI-web vs. usual care^2^ |  | 9.0 ± 3.5† |  | 8.0 ± 3.9* |  | 14.2 ± 7.7 |  |
| CCI-onsite vs. usual care^2^ |  | 12.6 ± 3.2§ |  | 9.2 ± 3.6† |  | 24.6 ± 7.4‡ |  |
| CCI-all vs. usual care^2^ |  | 10.9 ± 2.9‡ |  | 8.6 ± 3.2† |  | 19.3 ± 6.8† |  |
|  |  |  |  |  |  |  |  |
| **BMI (kg·m^-2^)** |  |  |  |  |  |  |  |
| All | 340 | 39.5 (8.6) | 253 | 39.1 (7.9) | 87 | 40.7 (10.4) | -1.5 ± 1.2 |
| CCI-all education^1^ | 257 | 40.4 (8.8) | 184 | 39.9 (7.9) | 73 | 41.8 (10.8) | -1.9 ± 1.4 |
| CCI-web^1^ | 121 | 39.4 (9.1) | 84 | 38.8 (7.8) | 37 | 40.6 (11.6) | -1.8 ± 2.1 |
| CCI-onsite^1^ | 136 | 41.4 (8.4) | 100 | 40.8 (7.9) | 36 | 43.1 (9.8) | -2.3 ± 1.8 |
| Usual care^1^ | 83 | 36.7 (7.3) | 69 | 37.1 (7.6) | 14 | 34.7 (4.8) | 2.5 ± 1.6 |
| CCI-web vs. CCI-onsite^2^ |  | -2.0 ± 1.1 |  | -2.0 ± 1.2 |  | -2.5 ± 2.5 |  |
| CCI-web vs. usual care^2^ |  | 2.6 ± 1.2* |  | 1.7 ± 1.3 |  | 5.9 ± 2.3† |  |
| CCI-onsite vs. usual care^2^ |  | 4.7 ± 1.1§ |  | 3.6 ± 1.2† |  | 8.4 ± 2.1§ |  |
| CCI-all vs. usual care^2^ |  | 3.7 ± 1.0‡ |  | 2.7 ± 1.1† |  | 7.2 ± 1.8§ |  |
|  |  |  |  |  |  |  |  |
| **Hemoglobin A1c (%)** |  |  |  |  |  |  |  |
| All | 349 | 7.61 (1.57) | 276 | 7.56 (1.52) | 73 | 7.79 (1.73) | -0.23 ± 0.22 |
| CCI-all education^1^ | 262 | 7.60 (1.50) | 204 | 7.49 (1.40) | 58 | 7.96 (1.79) | -0.47 ± 0.25 |
| CCI-web^1^ | 126 | 7.52 (1.41) | 98 | 7.43 (1.28) | 28 | 7.81 (1.78) | -0.37 ± 0.36 |
| CCI-onsite^1^ | 136 | 7.67 (1.59) | 106 | 7.55 (1.51) | 30 | 8.10 (1.82) | -0.56 ± 0.36 |
| Usual care^1^ | 87 | 7.64 (1.76) | 72 | 7.74 (1.82) | 15 | 7.14 (1.36) | 0.60 ± 0.41 |
| CCI-web vs. CCI-onsite^2^ |  | -0.15 ± 0.19 |  | -0.11 ± 0.20 |  | -0.30 ± 0.47 |  |
| CCI-web vs. usual care^2^ |  | -0.12 ± 0.23 |  | -0.31 ± 0.25 |  | 0.67 ± 0.48 |  |
| CCI-onsite vs. usual care^2^ |  | 0.03 ± 0.23 |  | -0.20 ± 0.26 |  | 0.97 ± 0.48* |  |
| CCI-all vs. usual care^2^ |  | -0.04 ± 0.21 |  | -0.25 ± 0.24 |  | 0.82 ± 0.42* |  |
|  |  |  |  |  |  |  |  |
| **Systolic blood pressure (mmHg)** |  |  |  |  |  |  |  |
| All | 339 | 131(14) | 254 | 132 (14) | 85 | 131 (14) | 0 ± 2 |
| CCI-all education^1^ | 260 | 132 (14) | 187 | 133 (15) | 73 | 130 (13) | 2 ± 2 |
| CCI-web^1^ | 124 | 133 (14) | 87 | 133 (15) | 37 | 131 (13) | 2 ± 3 |
| CCI-onsite^1^ | 136 | 131 (14) | 100 | 132 (14) | 36 | 130 (13) | 2 ± 3 |
| Usual care^1^ | 79 | 130 (14) | 67 | 129 (13) | 12 | 136 (17) | -7 ± 5 |
| CCI-web vs. CCI-onsite^2^ |  | 1 ± 2 |  | 1 ± 2 |  | 2 ± 3 |  |
| CCI-web vs. usual care^2^ |  | 3 ± 2 |  | 5 ± 2* |  | -4 ± 5 |  |
| CCI-onsite vs. usual care^2^ |  | 1 ± 2 |  | 3 ± 2 |  | -6 ± 6 |  |
| CCI-all vs. usual care^2^ |  | 2 ± 2 |  | 4 ± 2* |  | -5 ± 5 |  |
|  |  |  |  |  |  |  |  |
| **Diastolic blood pressure (mmHg)** |  |  |  |  |  |  |  |
| All | 339 | 82 (8) | 254 | 81 (8) | 85 | 84 (9) | -2 ± 1* |
| CCI-all education^1^ | 260 | 82 (8) | 187 | 82 (8) | 73 | 83 (9) | -2 ± 1 |
| CCI-web^1^ | 124 | 83 (9) | 87 | 82 (8) | 37 | 85 (10) | -4 ± 2* |
| CCI-onsite^1^ | 136 | 82 (8) | 100 | 82 (8) | 36 | 82 (7) | 0 ± 1 |
| Usual care^1^ | 79 | 82 (9) | 67 | 81 (8) | 12 | 87 (12) | -6 ± 4 |
| CCI-web vs. CCI-onsite^2^ |  | 1 ± 1 |  | 0 ± 1 |  | 4 ± 2 |  |
| CCI-web vs. usual care^2^ |  | 1 ± 1 |  | 0 ± 1 |  | -2 ± 4 |  |
| CCI-onsite vs. usual care^2^ |  | 0 ± 1 |  | 1 ± 1 |  | -5 ± 4 |  |
| CCI-all vs. usual care^2^ |  | 0 ± 1 |  | 0 ± 1 |  | -4 ± 4 |  |
|  |  |  |  |  |  |  |  |
| **ApoB (mg·dL-1)** |  |  |  |  |  |  |  |
| All | 327 | 106 (29) | 245 | 104 (28) | 82 | 110 (29) | -6 ± 4 |
| CCI-all education^1^ | 248 | 105 (29) | 186 | 103 (28) | 62 | 110 (31) | -6 ± 4 |
| CCI-web^1^ | 120 | 108 (30) | 88 | 103 (29) | 32 | 121 (30) | -18 ± 6† |
| CCI-onsite^1^ | 128 | 102 (27) | 98 | 104 (27) | 30 | 97 (26) | 6 ± 6 |
| Usual care^1^ | 79 | 107 (28) | 59 | 106 (30) | 20 | 111 (24) | -5 ± 7 |
| CCI-web vs. CCI-onsite^2^ |  | 6 ± 4 |  | -1 ± 4 |  | 24 ± 7‡ |  |
| CCI-web vs. usual care^2^ |  | 1 ± 4 |  | -3 ± 5 |  | 11 ± 7 |  |
| CCI-onsite vs. usual care^2^ |  | -5 ± 4 |  | -2 ± 5 |  | -13 ± 7 |  |
| CCI-all vs. usual care^2^ |  | -2 ± 4 |  | -2 ± 4 |  | -1 ± 7 |  |
|  |  |  |  |  |  |  |  |
| **ApoA1 (mg·dL-1)** |  |  |  |  |  |  |  |
| All | 327 | 146 (26) | 244 | 146 (27) | 83 | 146 (23) | 0 ± 3 |
| CCI-all education^1^ | 248 | 146 (28) | 185 | 146 (29) | 63 | 145 (23) | 1 ± 4 |
| CCI-web^1^ | 120 | 146 (29) | 88 | 145 (31) | 32 | 146 (20) | -1 ± 5 |
| CCI-onsite^1^ | 128 | 146 (27) | 97 | 146 (27) | 31 | 144 (25) | 3 ± 5 |
| Usual care^1^ | 79 | 149 (22) | 59 | 148 (21) | 20 | 150 (24) | -2 ± 6 |
| CCI-web vs. CCI-onsite^2^ |  | 0 ± 4 |  | -1 ± 4 |  | 3 ± 6 |  |
| CCI-web vs. usual care^2^ |  | -3 ± 4 |  | -3 ± 4 |  | -4 ± 6 |  |
| CCI-onsite vs. usual care^2^ |  | -3 ± 3 |  | -2 ± 4 |  | -6 ± 7 |  |
| CCI-all vs. usual care^2^ |  | -3 ± 3 |  | -2 ± 3 |  | -5 ± 6 |  |
|  |  |  |  |  |  |  |  |
| **ApoB/ApoA1 ratio** |  |  |  |  |  |  |  |
| All | 327 | 0.74 (0.23) | 244 | 0.73 (0.23) | 83 | 0.77 (0.23) | -0.04 ± 0.03 |
| CCI-all education^1^ | 248 | 0.74 (0.23) | 185 | 0.73 (0.23) | 63 | 0.78 (0.24) | -0.05 ± 0.03 |
| CCI-web^1^ | 120 | 0.77 (0.24) | 88 | 0.74 (0.24) | 32 | 0.84 (0.22) | -0.10 ± 0.05* |
| CCI-onsite^1^ | 128 | 0.72 (0.22) | 97 | 0.72 (0.22) | 31 | 0.71 (0.24) | 0.01 ± 0.05 |
| Usual care^1^ | 79 | 0.73 (0.23) | 59 | 0.73 (0.25) | 20 | 0.75 (0.19) | -0.03 ± 0.05 |
| CCI-web vs. CCI-onsite^2^ |  | 0.05 ± 0.03 |  | 0.02 ± 0.03 |  | 0.13 ± 0.06* |  |
| CCI-web vs. usual care^2^ |  | 0.03 ± 0.03 |  | 0.01 ± 0.04 |  | 0.09 ± 0.06 |  |
| CCI-onsite vs. usual care^2^ |  | -0.01 ± 0.03 |  | 0.00 ± 0.04 |  | -0.04 ± 0.06 |  |
| CCI-all vs. usual care^2^ |  | 0.01 ± 0.03 |  | 0.00 ± 0.04 |  | 0.03 ± 0.05 |  |
|  |  |  |  |  |  |  |  |
| **Triglycerides (mg·dL^-1^)** |  |  |  |  |  |  |  |
| All | 326 | 218 (236) | 245 | 224 (263) | 81 | 200 (119) | 24 ± 21 |
| CCI-all education^1^ | 247 | 197 (143) | 186 | 201 (153) | 61 | 187 (111) | 14 ± 18 |
| CCI-web^1^ | 120 | 190 (104) | 88 | 191 (106) | 32 | 186 (98) | 6 ± 21 |
| CCI-onsite^1^ | 127 | 204 (173) | 98 | 209 (185) | 29 | 188 (125) | 21 ± 30 |
| Usual care^1^ | 79 | 283 (401) | 59 | 297 (458) | 20 | 240 (138) | 57 ± 67 |
| CCI-web vs. CCI-onsite^2^ |  | -14 ± 18 |  | -18 ± 22 |  | -3 ± 29 |  |
| CCI-web vs. usual care^2^ |  | -93 ± 46* |  | -106 ± 61 |  | -55 ± 35 |  |
| CCI-onsite vs. usual care^2^ |  | -79 ± 48* |  | -88 ± 62 |  | -52 ± 39 |  |
| CCI-all vs. usual care^2^ |  | -86 ± 46* |  | -97 ± 61 |  | -54 ± 34* |  |
|  |  |  |  |  |  |  |  |
| **LDL-C (mg·dL^-1^)** |  |  |  |  |  |  |  |
| All | 302 | 102 (34) | 220 | 100 (34) | 82 | 108 (33) | -8 ± 4 |
| CCI-all education^1^ | 232 | 103 (33) | 172 | 100 (33) | 60 | 109 (33) | -9 ± 5 |
| CCI-web^1^ | 115 | 103 (36) | 83 | 97 (36) | 32 | 118 (31) | -21 ± 7† |
| CCI-onsite^1^ | 117 | 102 (30) | 89 | 103 (29) | 28 | 99 (33) | 34 ± 7 |
| Usual care^1^ | 70 | 102 (36) | 48 | 100 (38) | 22 | 104 (33) | -4 ± 9 |
| CCI-web vs. CCI-onsite^2^ |  | 1 ± 4 |  | -6 ± 5 |  | 19 ± 8* |  |
| CCI-web vs. usual care^2^ |  | 2 ± 5 |  | -3 ± 7 |  | 15 ± 9 |  |
| CCI-onsite vs. usual care^2^ |  | 0 ± 5 |  | 2 ± 6 |  | -5 ± 9 |  |
| CCI-all vs. usual care^2^ |  | 1 ± 5 |  | 0 ± 6 |  | 6 ± 8 |  |
|  |  |  |  |  |  |  |  |
| **HDL-C (mg·dL^-1^)** |  |  |  |  |  |  |  |
| All | 326 | 41 (13) | 245 | 41 (13) | 81 | 41 (12) | 0 ± 2 |
| CCI-all education^1^ | 247 | 42 (13) | 186 | 42 (14) | 61 | 42 (12) | 1 ± 2 |
| CCI-web^1^ | 120 | 42 (13) | 88 | 42 (14) | 32 | 42 (9) | 0 ± 2 |
| CCI-onsite^1^ | 127 | 42 (14) | 98 | 42 (14) | 29 | 41 (15) | 2 ± 3 |
| Usual care^1^ | 79 | 38 (11) | 59 | 37 (11) | 20 | 39 (11) | -2 ± 3 |
| CCI-web vs. CCI-onsite^2^ |  | 0 ± 2 |  | 0 ± 2 |  | 2 ± 3 |  |
| CCI-web vs. usual care^2^ |  | 6 ± 2† |  | 5 ± 2 |  | 3 ± 3 |  |
| CCI-onsite vs. usual care^2^ |  | 4 ± 2* |  | 5 ± 2† |  | 1 ± 4 |  |
| CCI-all vs. usual care^2^ |  | 5 ± 2† |  | 5 ± 2† |  | 2 ± 3 |  |
|  |  |  |  |  |  |  |  |
| **Triglycerides/HDL-C ratio** |  |  |  |  |  |  |  |
| All | 326 | 7.0 (13.1) | 245 | 7.4 (14.8) | 81 | 5.7 (4.8) | 1.7 ± 1.1 |
| CCI-all education^1^ | 247 | 5.9 (7.1) | 186 | 6.1 (7.9) | 61 | 5.1 (4.0) | 1.0 ± 0.8 |
| CCI-web^1^ | 120 | 5.3 (4.1) | 88 | 5.5 (4.4) | 32 | 4.7 (2.9) | 0.8 ± 0.7 |
| CCI-onsite^1^ | 127 | 6.4 (9.1) | 98 | 6.6 (10.0) | 29 | 5.6 (4.9) | 1.0 ± 1.4 |
| Usual care^1^ | 79 | 10.5 (23.2) | 59 | 11.5 (26.5) | 20 | 7.4 (6.7) | 4.1 ± 3.8 |
| CCI-web vs. CCI-onsite^2^ |  | -1.0 ± 0.9 |  | -1.1 ± 1.1 |  | -0.8 ± 1.0 |  |
| CCI-web vs. usual care^2^ |  | -5.2 ± 2.6* |  | -6.0 ± 3.5 |  | -2.7 ± 1.6 |  |
| CCI-onsite vs. usual care^2^ |  | -4.1 ± 2.7 |  | -4.9 ± 3.6 |  | -1.9 ± 1.8 |  |
| CCI-all vs. usual care^2^ |  | -4.6 ± 2.6 |  | -5.4 ± 3.5 |  | -2.3 ± 1.6 |  |
|  |  |  |  |  |  |  |  |
| **Large VLDL-P (nmol·L^-1^)** |  |  |  |  |  |  |  |
| All | 342 | 10 (9) | 271 | 10 (10) | 71 | 10 (8) | 0 ± 1 |
| CCI-all education^1^ | 259 | 10 (8) | 203 | 9 (8) | 56 | 10 (8) | 0 ± 1 |
| CCI-web^1^ | 124 | 9 (7) | 97 | 9 (7) | 27 | 10 (7) | -1 ± 2 |
| CCI-onsite^1^ | 135 | 10 (9) | 106 | 10 (9) | 29 | 10 (9) | 0 ± 2 |
| Usual care^1^ | 83 | 12 (12) | 68 | 12 (13) | 15 | 12 (9) | 0 ± 3 |
| CCI-web vs. CCI-onsite^2^ |  | 0 ± 1 |  | 0 ± 1 |  | 0 ± 2 |  |
| CCI-web vs. usual care^2^ |  | -3 ± 2 |  | -3 ± 2 |  | -2 ± 3 |  |
| CCI-onsite vs. usual care^2^ |  | -2 ± 2 |  | -2 ± 2 |  | -3 ± 3 |  |
| CCI-all vs. usual care^2^ |  | -2 ± 1 |  | -2 ± 2 |  | -2 ± 3 |  |
|  |  |  |  |  |  |  |  |
| **Total LDL-P (nmol·L^-1^)** |  |  |  |  |  |  |  |
| All | 342 | 1297 (476) | 271 | 1283 (477) | 71 | 1353 (467) | -70 ± 63 |
| CCI-all education^1^ | 259 | 1300 (465) | 203 | 1296 (476) | 56 | 1315 (427) | -19 ± 66 |
| CCI-web^1^ | 124 | 1336 (494) | 97 | 1312 (510) | 27 | 1424 (431) | -112 ± 98 |
| CCI-onsite^1^ | 135 | 1267 (435) | 106 | 1281 (444) | 29 | 1214 (404) | 67 ± 86 |
| Usual care^1^ | 83 | 1289 (511) | 68 | 1243 (484) | 15 | 1494 (590) | -251 ± 163 |
| CCI-web vs. CCI-onsite^2^ |  | 69 ± 58 |  | 31 ± 67 |  | 209 ± 112 |  |
| CCI-web vs. usual care^2^ |  | 47 ± 72 |  | 68 ± 78 |  | -71 ± 174 |  |
| CCI-onsite vs. usual care^2^ |  | -22 ± 67 |  | 38 ± 73 |  | -280 ± 170 |  |
| CCI-all vs. usual care^2^ |  | 11 ± 63 |  | 52 ± 68 |  | -179 ± 163 |  |
|  |  |  |  |  |  |  |  |
| **Small LDL-P (nmol·L^-1^)** |  |  |  |  |  |  |  |
| All | 342 | 761 (365) | 271 | 758 (367) | 71 | 772 (360) | -15 ± 48 |
| CCI-all education^1^ | 259 | 774 (377) | 203 | 778 (378) | 56 | 762 (376) | 16 ± 57 |
| CCI-web^1^ | 124 | 815 (404) | 97 | 810 (396) | 27 | 834 (440) | -25 ± 94 |
| CCI-onsite^1^ | 135 | 737 (348) | 106 | 748 (361) | 29 | 695 (298) | 54 ± 65 |
| Usual care^1^ | 83 | 719 (322) | 68 | 699 (326) | 15 | 812 (298) | -113 ± 86 |
| CCI-web vs. CCI-onsite^2^ |  | 78 ± 47 |  | 61 ± 53 |  | 140 ± 101 |  |
| CCI-web vs. usual care^2^ |  | 96 ± 51 |  | 111 ± 56* |  | 23 ± 114 |  |
| CCI-onsite vs. usual care^2^ |  | 18 ± 46 |  | 49 ± 53 |  | -117 ± 95 |  |
| CCI-all vs. usual care^2^ |  | 55 ± 42 |  | 79 ± 48 |  | -50 ± 92 |  |
|  |  |  |  |  |  |  |  |
| **LDL-particle size (nm)** |  |  |  |  |  |  |  |
| All | 342 | 20.31 (0.55) | 269 | 20.3 (0.55) | 73 | 20.33 (0.57) | -0.03 ± 0.07 |
| CCI-all education^1^ | 259 | 20.30 (0.55) | 201 | 20.3 (0.55) | 58 | 20.32 (0.56) | -0.02 ± 0.08 |
| CCI-web^1^ | 124 | 20.27 (0.56) | 95 | 20.26 (0.56) | 29 | 20.33 (0.58) | -0.07 ± 0.12 |
| CCI-onsite^1^ | 135 | 20.33 (0.54) | 106 | 20.33 (0.54) | 29 | 20.30 (0.55) | 0.03 ± 0.11 |
| Usual care^1^ | 83 | 20.33 (0.56) | 68 | 20.32 (0.55) | 15 | 20.38 (0.62) | -0.06 ± 0.17 |
| CCI-web vs. CCI-onsite^2^ |  | -0.05 ± 0.07 |  | -0.07 ± 0.08 |  | 0.02 ± 0.15 |  |
| CCI-web vs. usual care^2^ |  | -0.06 ± 0.08 |  | -0.06 ± 0.09 |  | -0.05 ± 0.19 |  |
| CCI-onsite vs. usual care^2^ |  | -0.01 ± 0.08 |  | 0.01 ± 0.08 |  | -0.08 ± 0.19 |  |
| CCI-all vs. usual care^2^ |  | -0.03 ± 0.07 |  | -0.03 ± 0.08 |  | 0.06 ± 0.17 |  |
|  |  |  |  |  |  |  |  |
| **Total HDL-P (µmol·L^-1^)** |  |  |  |  |  |  |  |
| All | 342 | 31.0 (6.2) | 271 | 31.3 (6.4) | 71 | 29.8 (5.6) | 1.6 ± 0.8* |
| CCI-all education^1^ | 259 | 31.3 (6.4) | 203 | 31.7 (6.4) | 56 | 30.0 (5.9) | 1.7 ± 0.9 |
| CCI-web^1^ | 124 | 31.5 (6.9) | 97 | 31.6 (7.0) | 27 | 31.0 (6.3) | 0.6 ± 1.4 |
| CCI-onsite^1^ | 135 | 31.2 (5.9) | 106 | 31.8 (5.9) | 29 | 29.1 (5.4) | 2.7 ± 1.2* |
| Usual care^1^ | 83 | 29.9 (5.8) | 68 | 30.2 (6.0) | 15 | 28.7 (4.6) | 1.5 ± 1.4 |
| CCI-web vs. CCI-onsite^2^ |  | 0.3 ± 0.8 |  | -0.2 ± 0.9 |  | 1.9 ± 1.6 |  |
| CCI-web vs. usual care^2^ |  | 1.6 ± 0.9 |  | 1.4 ± 1.0 |  | 2.3 ± 1.7 |  |
| CCI-onsite vs. usual care^2^ |  | 1.3 ± 0.8 |  | 1.6 ± 0.9 |  | 0.4 ± 1.6 |  |
| CCI-all vs. usual care^2^ |  | 1.4 ± 0.8 |  | 1.5 ± 0.9 |  | 1.3 ± 1.4 |  |
|  |  |  |  |  |  |  |  |
| **Large HDL-P (µmol·L^-1^)** |  |  |  |  |  |  |  |
| All | 342 | 4.1 (2.4) | 271 | 4.1 (2.4) | 71 | 4.2 (2.5) | 0.0 ± 0.3 |
| CCI-all education^1^ | 259 | 4.3 (2.5) | 203 | 4.2 (2.5) | 56 | 4.3 (2.6) | 0.0 ± 0.4 |
| CCI-web^1^ | 124 | 4.3 (2.6) | 97 | 4.3 (2.6) | 27 | 4.1 (2.5) | 0.2 ± 0.5 |
| CCI-onsite^1^ | 135 | 4.2 (2.5) | 106 | 4.2 (2.4) | 29 | 4.5 (2.7) | -0.3 ± 0.6 |
| Usual care^1^ | 83 | 3.8 (2.1) | 68 | 3.8 (2.14) | 15 | 3.8 (2.1) | 0.1 ± 0.6 |
| CCI-web vs. CCI-onsite^2^ |  | 0.0 ± 0.3 |  | 0.1 ± 0.4 |  | -0.4 ± 0.7 |  |
| CCI-web vs. usual care^2^ |  | 0.5 ± 0.3 |  | 0.5 ± 0.4 |  | 0.3 ± 0.7 |  |
| CCI-onsite vs. usual care^2^ |  | 0.4 ± 0.3 |  | 0.4 ± 0.4 |  | 0.7 ± 0.7 |  |
| CCI-all vs. usual care^2^ |  | 0.4 ± 0.3 |  | 0.4 ± 0.3 |  | 0.5 ± 0.6 |  |
|  |  |  |  |  |  |  |  |
| **LP-IR score** |  |  |  |  |  |  |  |
| All | 342 | 73 (17) | 271 | 73 (17) | 71 | 74 (16) | -2 ± 2 |
| CCI-all education^1^ | 259 | 72 (17) | 203 | 72 (18) | 56 | 73 (17) | -1 ± 3 |
| CCI-web^1^ | 124 | 72 (17) | 97 | 72 (18) | 27 | 75 (15) | -3 ± 3 |
| CCI-onsite^1^ | 135 | 72 (18) | 106 | 72 (17) | 29 | 71 (18) | 2 ± 4 |
| Usual care^1^ | 83 | 75 (16) | 68 | 74 (17) | 15 | 79 (13) | -5 ± 4 |
| CCI-web vs. CCI-onsite^2^ |  | 0 ± 2 |  | -1 ± 2 |  | 4 ± 5 |  |
| CCI-web vs. usual care^2^ |  | -3 ± 2 |  | -3 ± 3 |  | -5 ± 5 |  |
| CCI-onsite vs. usual care^2^ |  | -3 ± 2 |  | -2 ± 3 |  | -9 ± 5 |  |
| CCI-all vs. usual care^2^ |  | -3 ± 2 |  | -2 ± 2 |  | -70 ± 4 |  |
|  |  |  |  |  |  |  |  |
| **C-reactive protein (mg·L^-1^)** |  |  |  |  |  |  |  |
| All | 334 | 8.6 (13.2) | 263 | 9.0 (14.5) | 71 | 7.2 (6.5) | 1.9 ± 1.2 |
| CCI-all education^1^ | 249 | 8.5 (14.5) | 193 | 9.0 (16.1) | 56 | 7.0 (6.5) | 2.0 ± 1.5 |
| CCI-web^1^ | 126 | 9.4 (19.3) | 98 | 10.3 (21.7) | 28 | 6.1 (4.1) | 4.2 ± 2.3 |
| CCI-onsite^1^ | 123 | 7.7 (6.7) | 95 | 7.6 (6.2) | 28 | 7.9 (8.3) | -0.3 ± 1.7 |
| Usual care^1^ | 85 | 8.9 (8.6) | 70 | 9.1 (9.0) | 15 | 7.8 (6.6) | 1.4 ± 2.0 |
| CCI-web vs. CCI-onsite^2^ |  | 1.7 ± 1.8 |  | 2.7 ± 2.3 |  | -1.8 ± 1.7 |  |
| CCI-web vs. usual care^2^ |  | 0.5 ± 2.0 |  | 1.2 ± 2.4 |  | -1.7 ± 1.9 |  |
| CCI-onsite vs. usual care^2^ |  | -1.2 ± 1.1 |  | -1.5 ± 1.3 |  | 0.2 ± 2.3 |  |
| CCI-all vs. usual care^2^ |  | -0.3 ± 1.3 |  | -0.1 ± 1.6 |  | -0.7 ± 1.9 |  |
|  |  |  |  |  |  |  |  |
| **WBC** |  |  |  |  |  |  |  |
| All | 346 | 7.5 (2.1) | 276 | 7.4 (2.1) | 70 | 7.5 (2.1) | -0.1 ± 0.3 |
| CCI-all education^1^ | 260 | 7.2 (1.9) | 204 | 7.1 (1.8) | 56 | 7.7 (2.2) | -0.5 ± 0.3 |
| CCI-web^1^ | 125 | 7.3 (2.0) | 99 | 7.2 (1.9) | 26 | 7.5 (2.3) | -0.3 ± 0.5 |
| CCI-onsite^1^ | 135 | 7.2 (1.8) | 105 | 7.1 (1.7) | 30 | 7.8 (2.1) | -0.7 ± 0.4 |
| Usual care^1^ | 86 | 8.1 (2.4) | 72 | 8.3 (2.4) | 14 | 7.1 (1.9) | 1.2 ± 0.6* |
| CCI-web vs. CCI-onsite^2^ |  | 0.0 ± 0.2 |  | 0.1 ± 0.3 |  | -0.3 ± 0.6 |  |
| CCI-web vs. usual care^2^ |  | -0.9 ± 0.3† |  | -1.2 ± 0.4 |  | 0.4 ± 0.7 |  |
| CCI-onsite vs. usual care^2^ |  | -0.9 ± 0.3† |  | -1.3 ± 0.3‡ |  | 0.7 ± 0.6 |  |
| CCI-all vs. usual care^2^ |  | -0.9 ± 0.3† |  | -1.2 ± 0.3§ |  | 0.6 ± 0.6 |  |
|  |  |  |  |  |  |  |  |
| **10-year ASCVD risk (%)** |  |  |  |  |  |  |  |
| All | 270 | 11.3 (9.6) | 190 | 11.9 (9.7) | 80 | 9.9 (9.2) | 1.9 ± 1.2 |
| CCI-all education^1^ | 198 | 11.1 (9.1) | 135 | 12.1 (9.3) | 63 | 9.1 (8.5) | 3.0 ± 1.3* |
| CCI-web^1^ | 81 | 10.3 (6.9) | 53 | 11.5 (7.0) | 28 | 8.1 (6.2) | 3.3 ± 1.5 |
| CCI-onsite^1^ | 117 | 11.7 (10.4) | 82 | 12.5 (10.5) | 35 | 9.8 (10.0) | 2.7 ± 2.1 |
| Usual care^1^ | 72 | 11.8 (10.8) | 55 | 11.4 (10.8) | 17 | 13.2 (10.9) | -1.8 ± 3.0 |
| CCI-web vs. CCI-onsite^2^ |  | -1.4 ± 1.2 |  | -1.1 ± 1.5 |  | -1.7 ± 2.1 |  |
| CCI-web vs. usual care^2^ |  | -1.5 ± 1.5 |  | 0.1 ± 1.7 |  | -5.0 ± 2.9 |  |
| CCI-onsite vs. usual care^2^ |  | -0.1 ± 1.6 |  | 1.2 ± 1.9 |  | -3.3 ± 3.1 |  |
| CCI-all vs. usual care^2^ |  | -0.6 ± 1.4 |  | 0.8 ± 1.6 |  | -3.9 ± 2.8 |  |
|  |  |  |  |  |  |  |  |
|  |  |  |  |  |  |  |  |
| **CIMT-average (mm)** |  |  |  |  |  |  |  |
| All | 320 | 0.681 (0.110) | 212 | 0.688 (0.112) | 108 | 0.666 (0.103) | 0.023 ± 0.013 |
| CCI-all education^1^ | 236 | 0.681 (0.108) | 144 | 0.692 (0.113) | 92 | 0.662 (0.095) | 0.031 ± 0.014* |
| CCI-web^1^ | 115 | 0.670 (0.094) | 65 | 0.682 (0.101) | 50 | 0.656 (0.082) | 0.026 ± 0.017 |
| CCI-onsite^1^ | 121 | 0.690 (0.119) | 79 | 0.701 (0.122) | 42 | 0.669 (0.110) | 0.032 ± 0.022 |
| Usual care^1^ | 84 | 0.681 (0.116) | 68 | 0.680 (0.111) | 16 | 0.687 (0.141) | -0.007 ± 0.038 |
| CCI-web vs. CCI-onsite^2^ |  | -0.020 ± 0.014 |  | -0.019 ± 0.019 |  | -0.014 ± 0.021 |  |
| CCI-web vs. usual care^2^ |  | -0.011 ± 0.015 |  | 0.002 ± 0.018 |  | -0.031 ± 0.037 |  |
| CCI-onsite vs. usual care^2^ |  | 0.009 ± 0.017 |  | 0.021 ± 0.019 |  | -0.018 ± 0.039 |  |
| CCI-all vs. usual care^2^ |  | -0.001 ± 0.014 |  | 0.013 ± 0.016 |  | -0.025 ± 0.037 |  |
|  |  |  |  |  |  |  |  |
| **Statin (%)** |  |  |  |  |  |  |  |
| All | 349 | 52.2 ± 2.7 | 291 | 52.6 ± 2.9 | 58 | 50.0 ± 6.6 | 2.6 ± 7.2 |
| CCI-all education^1^ | 262 | 50.0 ± 3.1 | 218 | 51.8 ± 3.4 | 44 | 40.9 ± 7.4 | 10.9 ± 8.2 |
| CCI-web^1^ | 126 | 47.6 ± 4.5 | 104 | 51.0 ± 4.9 | 22 | 31.8 ± 9.9 | 19.1 ± 11.1 |
| CCI-onsite^1^ | 136 | 52.2 ± 4.3 | 114 | 52.6 ± 4.7 | 22 | 50.0 ± 10.7 | 2.6 ± 11.6 |
| Usual care^1^ | 87 | 58.6 ± 5.3 | 73 | 54.8 ± 5.8 | 14 | 78.6 ± 11.0 | -23.8 ± 12.4 |
| CCI-web vs. CCI-onsite^2^ |  | -4.6 ± 6.2 |  | -1.7 ± 6.8 |  | -18.2 ± 14.6 |  |
| CCI-web vs. usual care^2^ |  | -11.0 ± 6.9 |  | -3.8 ± 7.6 |  | -46.8 ± 14.8† |  |
| CCI-onsite vs. usual care^2^ |  | -6.4 ± 6.8 |  | -2.2 ± 7.5 |  | -28.6 ± 15.3 |  |
| CCI-all vs. usual care^2^ |  | -8.6 ± 6.1 |  | -3.0 ± 6.7 |  | -37.7 ± 13.2† |  |
|  |  |  |  |  |  |  |  |
| **Any antihypertensive medication (%)** |  |  |  |  |  |  |  |
| All | 349 | 63.6 ± 2.6 | 291 | 63.9 ± 2.8 | 58 | 62.1 ± 6.4 | 1.9 ± 7.0 |
| CCI-all education^1^ | 262 | 67.2 ± 2.9 | 218 | 68.4 ± 3.2 | 44 | 61.4 ± 7.3 | 7.0 ± 8.0 |
| CCI-web^1^ | 126 | 65.1 ± 4.3 | 104 | 65.4 ± 4.7 | 22 | 63.6 ± 10.3 | 1.8 ± 11.3 |
| CCI-onsite^1^ | 136 | 69.1 ± 4.0 | 114 | 71.1 ± 4.3 | 22 | 59.1 ± 10.5 | 12.0 ± 11.3 |
| Usual care^1^ | 87 | 52.9 ± 5.4 | 73 | 50.7 ± 5.9 | 14 | 64.3 ± 12.8 | -13.6 ± 14.1 |
| CCI-web vs. CCI-onsite^2^ |  | -4.0 ± 5.8 |  | -5.7 ± 6.3 |  | 4.6 ± 14.7 |  |
| CCI-web vs. usual care^2^ |  | 12.2 ± 6.8 |  | 14.7 ± 7.5* |  | -0.7 ± 16.4 |  |
| CCI-onsite vs. usual care^2^ |  | 16.2 ± 6.7* |  | 20.4 ± 7.2† |  | -5.2 ± 16.6 |  |
| CCI-all vs. usual care^2^ |  | 14.3 ± 6.1* |  | 17.7 ± 6.7† |  | -2.9 ± 14.8 |  |
|  |  |  |  |  |  |  |  |
| **ACE or ARB (%)** |  |  |  |  |  |  |  |
| All | 349 | 26.7 ± 2.4 | 291 | 25.1 ± 2.5 | 58 | 34.5 ± 6.2 | -9.4 ± 6.7 |
| CCI-all education^1^ | 262 | 29.4 ± 2.8 | 218 | 28.0 ± 3.0 | 44 | 36.4 ± 7.3 | -8.4 ± 7.9 |
| CCI-web^1^ | 126 | 32.5 ± 4.2 | 104 | 30.8 ± 4.5 | 22 | 40.9 ± 10.5 | -10.1 ± 11.4 |
| CCI-onsite^1^ | 136 | 26.5 ± 3.8 | 114 | 25.4 ± 4.1 | 22 | 31.8 ± 9.9 | -6.4 ± 10.7 |
| Usual care^1^ | 87 | 18.4 ± 4.2 | 73 | 16.4 ± 4.3 | 14 | 28.6 ± 12.1 | -12.1 ± 12.8 |
| CCI-web vs. CCI-onsite^2^ |  | 6.1 ± 5.6 |  | 5.3 ± 6.1 |  | 9.1 ± 14.4 |  |
| CCI-web vs. usual care^2^ |  | 14.2 ± 5.9* |  | 14.3 ± 6.3* |  | 12.3 ± 16.0 |  |
| CCI-onsite vs. usual care^2^ |  | 8.1 ± 5.6 |  | 9.0 ± 6.0 |  | 3.3 ± 15.6 |  |
| CCI-all vs. usual care^2^ |  | 11.0 ± 5.0* |  | 11.5 ± 5.3* |  | 7.8 ± 14.1 |  |
|  |  |  |  |  |  |  |  |
| **Diuretics (%)** |  |  |  |  |  |  |  |
| All | 349 | 38.1 ± 2.6 | 291 | 37.1 ± 2.8 | 58 | 43.1 ± 6.5 | -6.0 ± 7.1 |
| CCI-all education^1^ | 262 | 40.8 ± 3.0 | 218 | 41.3 ± 3.3 | 44 | 38.6 ± 7.3 | 2.7 ± 8.1 |
| CCI-web^1^ | 126 | 40.5 ± 4.4 | 104 | 41.4 ± 4.8 | 22 | 36.4 ± 10.3 | 5.0 ± 11.3 |
| CCI-onsite^1^ | 136 | 41.2 ± 4.2 | 114 | 41.2 ± 4.6 | 22 | 40.9 ± 10.5 | 0.3 ± 11.5 |
| Usual care^1^ | 87 | 29.9 ± 4.9 | 73 | 24.7 ± 5.0 | 14 | 57.1 ± 13.2 | -32.5 ± 14.2* |
| CCI-web vs. CCI-onsite^2^ |  | -0.7 ± 6.1 |  | 0.1 ± 6.7 |  | -4.6 ± 14.7 |  |
| CCI-web vs. usual care^2^ |  | 10.6 ± 6.6 |  | 16.7 ± 7.0* |  | -20.8 ± 16.7 |  |
| CCI-onsite vs. usual care^2^ |  | 11.3 ± 6.5 |  | 16.6 ± 6.8* |  | -16.2 ± 16.9 |  |
| CCI-all vs. usual care^2^ |  | 11.0 ± 5.8 |  | 16.6 ± 6.1† |  | -18.5 ± 15.1 |  |
|  |  |  |  |  |  |  |  |
| ^1^Mean and standard deviations for continuous variables, percents and standard errors for categorical variables | | | | | | | |
| ^2^Difference between means or percentages ± 1 standard error of the difference. Significant baseline difference between means or percentages are designated by the following symbols: *, 0.05 > P ≥ 0.01; †, 0.01 > P ≥ 0.001; ‡, 0.001 > P ≥ 0.0001; §, P < 0.0001 | | | | | | | |
