# Supplementary material for: Cardiovascular disease risk factor responses to a type 2 diabetes care model including nutritional ketosis induced by sustained carbohydrate restriction at 1 year: an open label, non-randomized, controlled study
Source: Cardiovasc Diabetol. 2018 May 1;17:56. doi: 10.1186/s12933-018-0698-8 (PMC5928595; doi:10.1186/s12933-018-0698-8)
Supplement: Supplementary file 2 — Additional file 2: Table S2. Details on 1-year biomarker changes for participants in the continuous care intervention group compared to usual care group. [file 12933_2018_698_MOESM2_ESM.docx]

| Supplementary Table 2. Details on 1-year biomarker changes for participants in the continuous care intervention group compared to usual care group | | | | | | | | | |
| --- | --- | --- | --- | --- | --- | --- | --- | --- | --- |
|  | **Completers** | | | | | | **All starters** | | |
|  |  |  |  |  |  |  | **(Dropouts imputed)^4^** | | |
|  | **N** |  | **Unadjusted** | | **Adjusted for baseline^3^** | | **Unadjusted** | | |
|  |  | **One Year Mean ±SE** | **Difference (SD) or ±SE** | **Signif-** | **Difference ±SE** | **Signif-** | **One Year Mean ±SE** | **Difference ±SE** | **Signif-** |
|  |  |  |  | **icance^5^** |  | **icance^5^** |  |  | **icance^5^** |
|  |  |  |  |  |  |  |  |  |  |
| **∆Weight-clinic (kg)** |  |  |  |  |  |  |  |  |  |
| CCI-all education^1^ | 184 | 101.2 ± 1.6 | -14.2 (10.3) | <10^-16^ | -13.8 ± 0.6 | <10^-16^ | 102.7 ± 1.5 | -13.8 ± 0.7 | <10^-16^ |
| CCI-web^1^ | 84 | 101.0 ± 2.5 | -13.8 (9.8) | <10^-16^ | -13.9 ± 0.9 | <10^-16^ | 101.0 ± 2.3 | -13.7 ± 1.0 | <10^-16^ |
| CCI-onsite^1^ | 100 | 101.3 ± 2.1 | -14.6 (10.7) | <10^-16^ | -13.7 ± 0.9 | <10^-16^ | 104.3 ± 2.0 | -13.9 ± 1.0 | <10^-16^ |
| Usual care^1^ | 69 | 106.8 ± 2.7 | 0.0 (5.9) | 0.95 | -1.1 ± 1.1 | 0.29 | 107.3 ± 2.6 | -0.2 ± 0.8 | 0.85 |
| CCI-web vs. CCI-onsite^2^ |  |  | 0.9 ± 1.5 | 0.57 | -0.2 ± 1.3 | 0.86 |  | 0.2 ± 1.4 | 0.89 |
| CCI-web vs. usual care^2^ |  |  | -13.8 ± 1.3 | <10^-16^ | -12.8 ± 1.4 | <10^-16^ |  | -13.6 ± 1.3 | <10^-16^ |
| CCI-onsite vs. usual care^2^ |  |  | -14.7 ± 1.3 | <10^-16^ | -12.6 ± 1.4 | <10^-16^ |  | -13.7 ± 1.3 | <10^-16^ |
| CCI-all vs. usual care^2^ |  |  | -14.3 ± 1.0 | <10^-16^ | -12.7 ± 1.3 | <10^-16^ |  | -13.7 ± 1.1 | <10^-16^ |
|  |  |  |  |  |  |  |  |  |  |
| **∆Hemoglobin A1c (%)** |  |  |  |  |  |  |  |  |  |
| CCI-all education^1^ | 204 | 6.2 ± 0.07 | -1.29 (1.32) | <10^-16^ | -1.32 ± 0.09 | <10^-16^ | 6.29 ± 0.07 | -1.30 ± 0.09 | <10^-16^ |
| CCI-web^1^ | 98 | 6.25 ± 0.09 | -1.18 (1.12) | <10^-16^ | -1.20 ± 0.13 | <10^-16^ | 6.31 ± 0.1 | -1.21 ± 0.11 | <10^-16^ |
| CCI-onsite^1^ | 106 | 6.15 ± 0.1 | -1.40 (1.47) | <10^-16^ | -1.43 ± 0.13 | <10^-16^ | 6.28 ± 0.1 | -1.39 ± 0.13 | <10^-16^ |
| Usual care^1^ | 72 | 7.94 ± 0.22 | 0.20 (1.35) | 0.21 | 0.22 ± 0.16 | 0.17 | 7.84 ± 0.19 | 0.20 ± 0.15 | 0.18 |
| CCI-web vs. CCI-onsite^2^ |  |  | 0.21 ± 0.18 | 0.24 | 0.22 ± 0.19 | 0.23 |  | 0.19 ± 0.17 | 0.28 |
| CCI-web vs. usual care^2^ |  |  | -1.38 ± 0.20 | 1.5x10^-12^ | -1.43 ± 0.21 | 1.1x10^-11^ |  | -1.40 ± 0.19 | 3.8x10^-14^ |
| CCI-onsite vs. usual care^2^ |  |  | -1.60 ± 0.21 | 8.7x10^-14^ | -1.65 ± 0.21 | 6.7x10^-15^ |  | -1.59 ± 0.20 | 6.7x10^-16^ |
| CCI-all vs. usual care^2^ |  |  | -1.49 ± 0.18 | 4.4x10^-16^ | -1.54 ± 0.19 | 4.4x10^-16^ |  | -1.50 ± 0.17 | <10^-16^ |
|  |  |  |  |  |  |  |  |  |  |
| **∆Systolic blood pressure (mmHg)** |  |  |  |  |  |  |  |  |  |
| CCI-all education^1^ | 187 | 126 ± 1 | -7 (16) | 1.3x10^-8^ | -7 ± 1 | 1.6x10^-7^ | 126 ± 1 | -6 ± 1 | 1.3x10^-8^ |
| CCI-web^1^ | 87 | 126 ± 1 | -7 (16) | 2.7x10^-5^ | -7 ± 2 | 5.2x10^-5^ | 126 ± 1 | -7 ± 2 | 2.3x10^-5^ |
| CCI-onsite^1^ | 100 | 126 ± 1 | -6 (16) | 0.0001 | -6 ± 2 | 0.0007 | 125 ± 1 | -6 ± 2 | 0.0001 |
| Usual care^1^ | 67 | 129 ± 2 | 0 (18) | 0.91 | 0 ± 2 | 0.83 | 129 ± 2 | -1 ± 2 | 0.67 |
| CCI-web vs. CCI-onsite^2^ |  |  | -1 ± 2 | 0.63 | -2 ± 2 | 0.55 |  | -1 ± 2 | 0.64 |
| CCI-web vs. usual care^2^ |  |  | -8 ± 3 | 0.006 | -7 ± 3 | 0.02 |  | -6 ± 3 | 0.02 |
| CCI-onsite vs. usual care^2^ |  |  | -6 ± 3 | 0.02 | -5 ± 3 | 0.06 |  | -5 ± 3 | 0.05 |
| CCI-all vs. usual care^2^ |  |  | -7 ±2 | 0.005 | -6 ± 3 | 0.02 |  | -5 ± 2 | 0.02 |
|  |  |  |  |  |  |  |  |  |  |
| **∆Diastolic blood pressure (mmHg)** |  |  |  |  |  |  |  |  |  |
| CCI-all education^1^ | 187 | 78 ± 1 | -4 (9) | 1.4x10^-7^ | -4 ± 1 | 6.2x10^-7^ | 79 ± 1 | -4 ± 1 | 7.2x10^-8^ |
| CCI-web^1^ | 87 | 78 ± 1 | -3 (10) | 0.002 | -3 ± 1 | 0.001 | 79 ± 1 | -4 ± 1 | 0.0002 |
| CCI-onsite^1^ | 100 | 78 ± 1 | -4 (9) | 1.6x10^-5^ | -4 ± 1 | 0.0001 | 78 ± 1 | -3 ± 1 | 0.0001 |
| Usual care^1^ | 67 | 81 ± 1 | 0 (10) | 0.92 | 0 ± 1 | 0.75 | 81 ± 1 | -1 ± 1 | 0.45 |
| CCI-web vs. CCI-onsite^2^ |  |  | 0 ± 1 | 0.75 | 0 ± 1 | 0.79 |  | 0 ± 1 | 0.71 |
| CCI-web vs. usual care^2^ |  |  | -3 ± 2 | 0.05 | -3 ± 2 | 0.07 |  | -3 ± 2 | 0.07 |
| CCI-onsite vs. usual care^2^ |  |  | -4 ± 2 | 0.02 | -3 ± 2 | 0.04 |  | -2 ± 1 | 0.11 |
| CCI-all vs. usual care^2^ |  |  | -3 ± 1 | 0.01 | -3 ± 1 | 0.03 |  | -3 ± 1 | 0.06 |
|  |  |  |  |  |  |  |  |  |  |
| **∆ApoB (mg·dL-1)** |  |  |  |  |  |  |  |  |  |
| CCI-all education^1^ | 186 | 103 ± 2 | -1 (24) | 0.69 | 0 ± 2 | 0.82 | 104 ± 2 | -2 ± 2 | 0.37 |
| CCI-web^1^ | 88 | 103 ± 3 | 0 (20) | 0.95 | 0 ± 3 | 0.93 | 105 ± 3 | -3 ± 3 | 0.28 |
| CCI-onsite^1^ | 98 | 103 ± 3 | -1 (27) | 0.66 | -1 ± 3 | 0.82 | 102 ± 3 | -1 ± 3 | 0.82 |
| Usual care^1^ | 59 | 107 ± 5 | 2 (37) | 0.75 | 1 ± 4 | 0.9 | 106 ± 4 | 0 ± 4 | 0.95 |
| CCI-web vs. CCI-onsite^2^ |  |  | 1 ± 3 | 0.76 | 0 ± 4 | 0.92 |  | -2 ± 4 | 0.56 |
| CCI-web vs. usual care^2^ |  |  | -2 ± 5 | 0.75 | -1 ± 5 | 0.87 |  | -3 ± 5 | 0.53 |
| CCI-onsite vs. usual care^2^ |  |  | -3 ± 6 | 0.62 | -1 ± 5 | 0.81 |  | -1 ± 5 | 0.86 |
| CCI-all vs. usual care^2^ |  |  | -2 ± 5 | 0.66 | -1 ± 5 | 0.83 |  | -2 ± 5 | 0.67 |
|  |  |  |  |  |  |  |  |  |  |
| **∆ApoA1 (mg·dL-1)** |  |  |  |  |  |  |  |  |  |
| CCI-all education^1^ | 185 | 160 ± 3 | 14 (24) | 8.9x10^-16^ | 14 ± 2 | 4.4x10^-16^ | 160 ± 2 | 14 ± 2 | <10^-16^ |
| CCI-web^1^ | 88 | 157 ± 4 | 12 (27) | 2.2x10^-5^ | 12 ± 2 | 3.5x10^-7^ | 158 ± 3 | 12 ± 3 | 1.3x10^-6^ |
| CCI-onsite^1^ | 97 | 163 ± 3 | 16 (22) | 9.7x10^-14^ | 15 ± 2 | 2.0x10^-10^ | 162 ± 3 | 16 ± 2 | 1.3x10^-13^ |
| Usual care^1^ | 59 | 1445 ± 3 | -3 (19) | 0.18 | -2 ± 3 | 0.55 | 147 ± 3 | -2 ± 3 | 0.37 |
| CCI-web vs. CCI-onsite^2^ |  |  | -4 ± 4 | 0.23 | -3 ± 3 | 0.43 |  | -4 ± 3 | 0.25 |
| CCI-web vs. usual care^2^ |  |  | 15 ± 4 | 4.0x10^-5^ | 14 ± 4 | 0.0003 |  | 15 ± 4 | 7.0x10^-5^ |
| CCI-onsite vs. usual care^2^ |  |  | 20 ± 3 | 1.9x10^-9^ | 17 ± 4 | 3.5x10^-5^ |  | 19 ± 4 | 8.7x10^-8^ |
| CCI-all vs. usual care^2^ |  |  | 18 ± 3 | 4.7x10^-9^ | 16 ± 4 | 2.2x10^-5^ |  | 17 ± 3 | 1.4x10^-7^ |
|  |  |  |  |  |  |  |  |  |  |
| **∆ ApoB/ApoA1** |  |  |  |  |  |  |  |  |  |
| CCI-all education^1^ | 185 | 0.67 ± 0.02 | -0.06 (0.17) | 1.8x10^-6^ | -0.06 ± 0.02 | 0.003 | 0.67 ± 0.02 | -0.07 ± 0.01 | 1.9x10^-7^ |
| CCI-web^1^ | 88 | 0.68 ± 0.02 | -0.06 (0.16) | 0.0006 | -0.06 ± 0.02 | 0.003 | 0.69 ± 0.02 | -0.08 ± 0.02 | 4.3x10^-5^ |
| CCI-onsite^1^ | 97 | 0.66 ± 0.02 | -0.06 (0.19) | 0.0009 | -0.06 ± 0.02 | 0.003 | 0.66 ± 0.02 | -0.07 ± 0.02 | 0.0008 |
| Usual care^1^ | 59 | 0.76 ± 0.04 | 0.03 (0.29) | 0.42 | 0.02 ± 0.03 | 0.5 | 0.74 ± 0.03 | 0.02 ± 0.03 | 0.58 |
| CCI-web vs. CCI-onsite^2^ |  |  | 0.01 ± 0.03 | 0.84 | 0.00 ± 0.03 | 0.9 |  | -0.01 ± 0.03 | 0.70 |
| CCI-web vs. usual care^2^ |  |  | -0.09 ± 0.04 | 0.03 | -0.08 ± 0.04 | 0.02 |  | -0.09 ± 0.04 | 0.01 |
| CCI-onsite vs. usual care^2^ |  |  | -0.09 ± 0.04 | 0.03 | -0.08 ± 0.04 | 0.04 |  | -0.08 ± 0.04 | 0.03 |
| CCI-all vs. usual care^2^ |  |  | -0.09 ± 0.04 | 0.02 | -0.08 ± 0.03 | 0.02 |  | -0.09 ± 0.03 | 0.01 |
|  |  |  |  |  |  |  |  |  |  |
| **∆Triglycerides (mg·dL^-1^)** |  |  |  |  |  |  |  |  |  |
| CCI-all education^1^ | 186 | 151 ± 11 | -49 (168) | 5.6x10^-5^ | -50 ± 16 | 0.001 | 148 ± 12 | -48 ± 13 | <10^-16^ |
| CCI-web^1^ | 88 | 159 ± 17 | -32 (159) | 0.06 | -30 ± 22 | 0.18 | 153 ± 17 | -35 ± 18 | 1.3x10^-7^ |
| CCI-onsite^1^ | 98 | 144 ± 14 | -65 (174) | 0.0002 | -69 ± 22 | 0.002 | 143 ± 156 | -59 ± 18 | 1.9x10^-12^ |
| Usual care^1^ | 59 | 327 ± 65 | 30 (301) | 0.44 | 31 ± 29 | 0.27 | 305 ± 48 | 28 ± 32 | 0.43 |
| CCI-web vs. CCI-onsite^2^ |  |  | 34 ± 24 | 0.17 | 39 ± 31 | 0.22 |  | 24 ± 25 | 0.34 |
| CCI-web vs. usual care^2^ |  |  | -62 ± 43 | 0.15 | -63 ± 36 | 0.08 |  | -63 ± 37 | 8.7x10^-5^ |
| CCI-onsite vs. usual care^2^ |  |  | -95 ± 43 | 0.03 | 102 ± 37 | 0.007 |  | -88 ± 37 | 1.2x10^-6^ |
| CCI-all vs. usual care^2^ |  |  | -80 ± 41 | 0.05 | -81 ± 33 | 0.02 |  | -76 ± 35 | 9.9x10^-7^ |
|  |  |  |  |  |  |  |  |  |  |
| **∆LDL-C (mg·dL^-1^)** |  |  |  |  |  |  |  |  |  |
| CCI-all education^1^ | 172 | 111 ± 3 | 11 (33) | 7.7x10^-6^ | 11 ± 3 | 2.6x10^-5^ | 113 ± 3 | 10 ± 2 | 4.9x10^-5^ |
| CCI-web^1^ | 83 | 109 ± 4 | 12 (33) | 0.002 | 12 ± 4 | 0.001 | 112 ± 4 | 9 ± 4 | 0.02 |
| CCI-onsite^1^ | 89 | 113 ± 4 | 10 (31) | 0.002 | 10 ± 4 | 0.007 | 114 ± 4 | 11 ± 3 | 0.0008 |
| Usual care^1^ | 48 | 90 ± 4 | -11 (38) | 0.05 | -11 ± 5 | 0.03 | 90 ± 5 | -11 ± 5 | 0.02 |
| CCI-web vs. CCI-onsite^2^ |  |  | 1 ± 5 | 0.8 | 2 ± 5 | 0.67 |  | -3 ± 5 | 0.59 |
| CCI-web vs. usual care^2^ |  |  | 22 ± 7 | 0.0006 | 23 ± 6 | 0.0004 |  | 20 ± 6 | 0.001 |
| CCI-onsite vs. usual care^2^ |  |  | 21 ± 6 | 0.0009 | 20 ± 6 | 0.002 |  | 22 ± 6 | 0.0001 |
| CCI-all vs. usual care^2^ |  |  | 22 ± 6 | 0.0003 | 22 ± 6 | 0.0002 |  | 21 ± 5 | 9.9x10^-5^ |
|  |  |  |  |  |  |  |  |  |  |
| **∆HDL-C (mg·dL^-1^)** |  |  |  |  |  |  |  |  |  |
| CCI-all education^1^ | 186 | 50 ± 1 | 8 (12) | <10^-16^ | 7 ± 1 | <10^-16^ | 50 ± 1 | 8 ± 1 | <10^-16^ |
| CCI-web^1^ | 88 | 49 ± 2 | 6 (13) | 2.9x10^-6^ | 6 ± 1 | 7.3x10^-8^ | 49 ± 2 | 7 ± 1 | 2.7x10^-8^ |
| CCI-onsite^1^ | 98 | 51 ± 2 | 9 (11) | 7.1x10^-15^ | 8 ± 1 | 5.3x10^-12^ | 51 ± 1 | 8 ± 1 | 2.0x10^-14^ |
| Usual care^1^ | 59 | 35 ± 2 | -2 (9) | 0.15 | -1 ± 2 | 0.69 | 37 ± 2 | -1 ± 1 | 0.41 |
| CCI-web vs. CCI-onsite^2^ |  |  | -2 ± 2 | 0.18 | -2 ± 2 | 0.36 |  | -2 ± 2 | 0.32 |
| CCI-web vs. usual care^2^ |  |  | 8 ± 2 | 7.1x10^-6^ | 7 ± 2 | 0.0003 |  | 8 ± 2 | 9.6x10^-7^ |
| CCI-onsite vs. usual care^2^ |  |  | 10 ± 2 | 1.1x10^-10^ | 9 ± 2 | 1.3x10^-5^ |  | 10 ± 2 | 2.1x10^-8^ |
| CCI-all vs. usual care^2^ |  |  | 9 ± 1 | 1.7x10^-10^ | 8 ± 2 | 9.9x10^-6^ |  | 9 ± 2 | 1.2x10^-8^ |
|  |  |  |  |  |  |  |  |  |  |
| **Triglycerides/HDL-C ratio** |  |  |  |  |  |  |  |  |  |
| CCI-all education^1^ | 186 | 4.3 ± 0.6 | -1.8 (9.4) | <10^-16^ | -1.9 ± 0.9 | <10^-16^ | 4.1 ± 0.6 | -1.6 ± 0.7 | <10^-16^ |
| CCI-web^1^ | 88 | 4.7 ± 1.0 | -0.8 (10.0) | 4.1x10^-7^ | -0.6 ± 1.2 | 5.5x10^-9^ | 4.3 ± 1.0 | -0.9 ± 1.0 | 5.1x10^-8^ |
| CCI-onsite^1^ | 98 | 3.9 ± 0.8 | -2.7 (8.9) | <10^-16^ | -3.1 ± 1.2 | 6.1x10^-15^ | 3.9 ± 0.8 | -2.4 ± 0.9 | 8.9x10^-16^ |
| Usual care^1^ | 59 | 12.5 ± 2.7 | 0.9 (16.1) | 0.1 | 1.2 ± 1.6 | 0.16 | 11.2 ± 2.1 | 1.0 ± 1.7 | 0.24 |
| CCI-web vs. CCI-onsite^2^ |  |  | 1.9 ± 1.4 | 0.12 | 2.5± 1.7 | 0.08 |  | 1.5 ± 1.4 | 0.28 |
| CCI-web vs. usual care^2^ |  |  | -1.8 ± 2.3 | 3.3x10^-6^ | -2.0 ± 2.0 | 1.5x10^-5^ |  | -1.8 ± 2.0 | 3.1x10^-6^ |
| CCI-onsite vs. usual care^2^ |  |  | -3.7 ± 2.3 | 6.4x10^-11^ | -4.4 ± 2.0 | 3.8 x10^-7^ |  | -3.3 ± 2.0 | 6.0x10^-9^ |
| CCI-all vs. usual care^2^ |  |  | -2.8 ± 2.2 | 3.1x10^-10^ | -3.1 ± 1.8 | 5.5x10^-7^ |  | -2.6 ± 1.8 | 4.5x10^-9^ |
|  |  |  |  |  |  |  |  |  |  |
| **∆Large VLDL-P (nmol·L^-1^)** |  |  |  |  |  |  |  |  |  |
| CCI-all education^1^ | 203 | 6 ± 1 | -4 (7) | 5.6x10^-15^ | -4 ± 1 | 1.6x10^-14^ | 6 ± 1 | -4 ± 1 | 4.2x10^-15^ |
| CCI-web^1^ | 97 | 6 ± 1 | -3 (7) | 6.4x10^-6^ | -3 ± 1 | 3.1x10^-5^ | 6 ± 1 | -3 ± 1 | 2.2x10^-5^ |
| CCI-onsite^1^ | 106 | 5 ± 1 | -4 (7) | 8.6x10^-11^ | -5 ± 1 | 3.4x10^-11^ | 6 ± 1 | -4 ± 1 | 3.7x10^-10^ |
| Usual care^1^ | 68 | 12 ± 2 | 0 (8) | 0.71 | 0 ± 1 | 0.60 | 12 ± 1 | 0 ± 1 | 0.77 |
| CCI-web vs. CCI-onsite^2^ |  |  | 1 ± 1 | 0.17 | 2 ± 1 | 0.1 |  | 1 ± 1 | 0.32 |
| CCI-web vs. usual care^2^ |  |  | -3 ± 1 | 0.02 | -3 ± 1 | 0.03 |  | -3 ± 1 | 0.009 |
| CCI-onsite vs. usual care^2^ |  |  | -4 ± 1 | 0.0005 | -4 ± 1 | 0.0005 |  | -4 ± 1 | 0.0005 |
| CCI-all vs. usual care^2^ |  |  | -3 ± 1 | 0.001 | -3 ± 1 | 0.002 |  | -3 ± 1 | 0.0007 |
|  |  |  |  |  |  |  |  |  |  |
| **∆Total LDL-P (nmol·L^-1^)** |  |  |  |  |  |  |  |  |  |
| CCI-all education^1^ | 203 | 1234 ± 30 | -62 (375) | 0.02 | -57 ± 29 | 0.05 | 1235 ± 29 | -64 ± 26 | 0.02 |
| CCI-web^1^ | 97 | 1229 ± 43 | -82 (383) | 0.03 | -81 ± 42 | 0.05 | 1244 ± 41 | -91 ± 39 | 0.02 |
| CCI-onsite^1^ | 106 | 1238 ± 43 | -43 (370) | 0.23 | -34 ± 41 | 0.40 | 1228 ± 41 | -38 ± 36 | 0.29 |
| Usual care^1^ | 68 | 1196 ± 60 | -47 (491) | 0.43 | -67 ± 53 | 0.21 | 1231 ± 57 | -57 ± 56 | 0.31 |
| CCI-web vs. CCI-onsite^2^ |  |  | -39 ± 53 | 0.46 | -47 ± 58 | 0.42 |  | -53 ± 53 | 0.31 |
| CCI-web vs. usual care^2^ |  |  | -35 ± 71 | 0.62 | -13 ± 68 | 0.85 |  | -34 ± 68 | 0.61 |
| CCI-onsite vs. usual care^2^ |  |  | 4 ± 70 | 0.96 | 34 ± 69 | 0.62 |  | 19 ± 66 | 0.78 |
| CCI-all vs. usual care^2^ |  |  | -15 ± 65 | 0.82 | 10 ± 62 | 0.87 |  | -7 ± 62 | 0.91 |
|  |  |  |  |  |  |  |  |  |  |
| **∆Small LDL-P (nmol·L^-1^)** |  |  |  |  |  |  |  |  |  |
| CCI-all education^1^ | 203 | 614 ± 22 | -164 (332) | 2.2x10^-12^ | -161 ± 24 | 4.1x10^-11^ | 613 ± 21 | -161 ± 23 | 1.2x10^-12^ |
| CCI-web^1^ | 97 | 623 ± 29 | -186 (354) | 2.3x10^-7^ | -188 ± 35 | 7.9x10^-8^ | 624 ± 30 | -190 ± 35 | 4.6x10^-9^ |
| CCI-onsite^1^ | 106 | 605 ± 32 | -143 (311) | 2.1x10^-6^ | -136 ± 34 | 6.5x10^-5^ | 603 ± 31 | -134 ± 30 | 6.3x10^-6^ |
| Usual care^1^ | 68 | 724 ± 44 | 25 (370) | 0.57 | 16 ± 45 | 0.71 | 740 ± 41 | 18 ± 42 | 0.67 |
| CCI-web vs. CCI-onsite^2^ |  |  | -43 ± 47 | 0.36 | -52 ± 49 | 0.29 |  | -56 ± 46 | 0.22 |
| CCI-web vs. usual care^2^ |  |  | -212 ± 57 | 0.0002 | -203 ± 57 | 0.0005 |  | -208 ± 55 | 0.0001 |
| CCI-onsite vs. usual care^2^ |  |  | -169 ± 54 | 0.002 | -151 ± 57 | 0.009 |  | -152 ± 52 | 0.003 |
| CCI-all vs. usual care^2^ |  |  | -189 ± 51 | 0.0002 | -177 ± 52 | 0.0007 |  | -179 ± 48 | 0.0002 |
|  |  |  |  |  |  |  |  |  |  |
| **∆LDL-particle size (nm)** |  |  |  |  |  |  |  |  |  |
| CCI-all education^1^ | 201 | 20.53 ± 0.04 | 0.23 (0.54) | 1.7x10^-9^ | 0.23 ± 0.04 | 8.9x10^-9^ | 20.53 ± 0.04 | 0.23 ± 0.04 | 6.0x10^-10^ |
| CCI-web^1^ | 95 | 20.5 ± 0.06 | 0.24 (0.51) | 4.6x10^-6^ | 0.24 ± 0.06 | 6.3x10^-5^ | 20.52 ± 0.06 | 0.24 ± 0.05 | 5.2x10^-5^ |
| CCI-onsite^1^ | 106 | 20.55 ± 0.05 | 0.22 (0.57) | 6.7x10^-5^ | 0.22 ± 0.05 | 1.1x10^-5^ | 20.54 ± 0.05 | 0.22 ± 0.05 | 3.0x10^-5^ |
| Usual care^1^ | 68 | 20.25 ± 0.07 | -0.08 (0.53) | 0.24 | -0.08 ± 0.07 | 0.25 | 20.25 ± 0.07 | -0.07 ± 0.06 | 0.25 |
| CCI-web vs. CCI-onsite^2^ |  |  | 0.02 ±0.08 | 0.76 | 0.03 ± 0.08 | 0.73 |  | 0.02 ± 0.07 | 0.8 |
| CCI-web vs. usual care^2^ |  |  | 0.32 ±0.08 | 0.0001 | 0.32 ± 0.09 | 0.0004 |  | 0.31 ± 0.08 | 0.0001 |
| CCI-onsite vs. usual care^2^ |  |  | 0.29 ±0.08 | 0.0005 | 0.30 ± 0.09 | 0.001 |  | 0.29 ± 0.08 | 0.0004 |
| CCI-all vs. usual care^2^ |  |  | 0.3 ±0.07 | 4.4x10^-5^ | 0.31 ± 0.08 | 0.0002 |  | 0.3 ± 0.07 | 3.8x10^-15^ |
|  |  |  |  |  |  |  |  |  |  |
| **∆Total HDL-P (µmol·L^-1^)** |  |  |  |  |  |  |  |  |  |
| CCI-all education^1^ | 203 | 33.2 ± 0.5 | 1.5 (4.9) | 1.2x10^-5^ | 1.5 ± 0.4 | 2.1x10^-5^ | 32.8 ± 0.4 | 1.5 ± 0.3 | 5.6x10^-6^ |
| CCI-web^1^ | 97 | 32.9 ± 0.7 | 1.3 (5.2) | 0.01 | 1.3 ± 0.5 | 0.01 | 32.6 ± 0.6 | 1.2 ± 0.5 | 0.02 |
| CCI-onsite^1^ | 106 | 33.5 ± 0.7 | 1.7 (4.7) | 0.0002 | 1.7 ± 0.5 | 0.0006 | 33.0 ± 0.6 | 1.8 ± 0.5 | 6.5x10^-5^ |
| Usual care^1^ | 68 | 29.4 ± 0.8 | -0.8 (4.7) | 0.15 | -0.8 ± 0.6 | 0.23 | 29.2 ± 0.7 | -0.7 ± 0.6 | 0.23 |
| CCI-web vs. CCI-onsite^2^ |  |  | -0.4 ± 0.7 | 0.55 | -0.4 ± 0.7 | 0.54 |  | -0.6 ± 0.7 | 0.40 |
| CCI-web vs. usual care^2^ |  |  | 2.1 ± 0.8 | 0.006 | 2.1 ± 0.8 | 0.01 |  | 1.9 ± 0.8 | 0.01 |
| CCI-onsite vs. usual care^2^ |  |  | 2.5 ± 0.7 | 0.0005 | 2.6 ± 0.8 | 0.003 |  | 2.5 ± 0.7 | 0.0006 |
| CCI-all vs. usual care^2^ |  |  | 2.3 ± 0.7 | 0.0004 | 2.3 ± 0.7 | 0.003 |  | 2.2 ± 0.7 | 0.0008 |
|  |  |  |  |  |  |  |  |  |  |
| **∆Large HDL-P (µmol·L^-1^)** |  |  |  |  |  |  |  |  |  |
| CCI-all education^1^ | 203 | 5.3 ± 0.2 | 1.0 (2.2) | 2.5x10^-11^ | 1.0 ± 0.2 | 4.1x10^-11^ | 5.3 ± 0.2 | 1 ± 0.15 | 1.2x10^-11^ |
| CCI-web^1^ | 97 | 5.1 ± 0.3 | 0.8 (2.2) | 0.0006 | 0.7 ± 0.2 | 0.0004 | 5.1 ± 0.3 | 0.75 ± 0.22 | 0.0007 |
| CCI-onsite^1^ | 106 | 5.4 ± 0.3 | 1.3 (2.1) | 1.5x10^-9^ | 1.2 ± 0.2 | 1.1x10^-9^ | 5.4 ± 0.3 | 1.24 ± 0.2 | 9.7x10^-10^ |
| Usual care^1^ | 68 | 3.9 ± 0.3 | 0.1 (1.6) | 0.69 | 0.2 ± 0.3 | 0.44 | 3.9 ± 0.3 | 0.07 ± 0.22 | 0.74 |
| CCI-web vs. CCI-onsite^2^ |  |  | -0.5 ±0.3 | 0.11 | -0.5 ± 0.3 | 0.11 |  | -0.49 ± 0.3 | 0.10 |
| CCI-web vs. usual care^2^ |  |  | 0.7 ±0.3 | 0.02 | 0.6 ± 0.3 | 0.11 |  | 0.68 ± 0.31 | 0.03 |
| CCI-onsite vs. usual care^2^ |  |  | 1.2 ±0.3 | 4.2x10^-5^ | 1.0 ± 0.3 | 0.003 |  | 1.17 ± 0.3 | 9.1x10^-5^ |
| CCI-all vs. usual care^2^ |  |  | 0.9 ±0.3 | 0.0002 | 0.8 ± 0.3 | 0.01 |  | 0.93 ± 0.26 | 0.0004 |
|  |  |  |  |  |  |  |  |  |  |
| **∆LP-IR score** |  |  |  |  |  |  |  |  |  |
| CCI-all education^1^ | 203 | 58 ± 2 | -14 (18) | <10^-16^ | -14 ± 1 | <10^-16^ | 58 ± 1 | -14 ± 1 | <10^-16^ |
| CCI-web^1^ | 97 | 59 ± 2 | -13 (18) | 2.1x10^-12^ | -13 ± 2 | 9.7x10^-13^ | 59 ± 2 | -13 ± 2 | 6.6x10^-14^ |
| CCI-onsite^1^ | 106 | 57 ± 2 | -15 (17) | <10^-16^ | -15 ± 2 | <10^-16^ | 57 ± 2 | -15 ± 2 | <10^-16^ |
| Usual care^1^ | 68 | 74 ± 2 | -1 (16) | 0.73 | -2 ± 2 | 0.41 | 75 ± 2 | -1 ± 2 | 0.74 |
| CCI-web vs. CCI-onsite^2^ |  |  | 2 ± 3 | 0.39 | 2 ± 2 | 0.84 |  | 1 ± 2 | 0.59 |
| CCI-web vs. usual care^2^ |  |  | -12 ± 3 | 3.5x10^-6^ | -11 ± 3 | 0.0002 |  | -13 ± 3 | 1.7x10^-6^ |
| CCI-onsite vs. usual care^2^ |  |  | -14 ± 3 | 1.4x10^-8^ | -13 ± 3 | 9.0x10^-6^ |  | -14 ± 3 | 4.7x10^-8^ |
| CCI-all vs. usual care^2^ |  |  | -13 ± 2 | 3.8x10^-9^ | -12 ± 23 | 6.2x10^-6^ |  | -13 ± 2 | 6.2x10^-9^ |
|  |  |  |  |  |  |  |  |  |  |
| **∆C-reactive protein (mg·L^-1^)** |  |  |  |  |  |  |  |  |  |
| CCI-all education^1^ | 193 | 5.7 ± 0.5 | -3.3 (13.4) | <10^-8^ | -3.1 ± 1.0 | <10^-16^ | 5.6 ± 0.6 | -3.6 ± 1.1 | <10^-16^ |
| CCI-web^1^ | 98 | 5.9 ± 0.7 | -4.5 (17.6) | 5.9x10^-11^ | -4.2 ± 1.3 | 1.5x10^-10^ | 5.7 ± 0.8 | -3.7 ± 1.5 | 6.1x10^-11^ |
| CCI-onsite^1^ | 95 | 5.4 ± 0.7 | -2.2 (6.5) | 3.1x10^-11^ | -1.9 ± 1.4 | 4.7x10^-12^ | 5.4 ± 0.8 | -3.6 ± 1.5 | 7.8x10^-13^ |
| Usual care^1^ | 70 | 10.4 ± 1.8 | 1.3 (13.3) | 0.94 | 0.9 ± 1.7 | 0.88 | 10.3 ± 1.6 | 1.3 ± 1.5 | 0.93 |
| CCI-web vs. CCI-onsite^2^ |  |  | -2.3 ± 1.9 | 0.44 | -2.3 ± 1.9 | 0.62 |  | -0.1 ± 2.1 | 0.43 |
| CCI-web vs. usual care^2^ |  |  | -5.8 ± 2.4 | 4.7x10^-5^ | -5.1 ± 2.2 | 3.5x10^-5^ |  | -4.8 ± 2.1 | 4.9x10^-5^ |
| CCI-onsite vs. usual care^2^ |  |  | -3.5 ± 1.7 | 6.7x10^-6^ | -2.8 ± 2.2 | 8.2x10^-6^ |  | -4.9 ± 2.1 | 3.4x10^-6^ |
| CCI-all vs. usual care^2^ |  |  | -4.7 ±1.9 | 1.2x10^-6^ | -4.0 ± 2.0 | 3.0x10^-5^ |  | -4.9 ± 1.8 | 9.3x10^-7^ |
|  |  |  |  |  |  |  |  |  |  |
| **∆WBC (k·mm^-3^)** |  |  |  |  |  |  |  |  |  |
| CCI-all education^1^ | 204 | 6.5 ± 0.1 | -0.7 (1.4) | 2.1x10^-11^ | -0.7 ± 0.1 | 2.1x10^-11^ | 6.6 ± 0.1 | -0.7± 0.1 | 3.2x10^-11^ |
| CCI-web^1^ | 99 | 6.5 ± 0.2 | -0.7 (1.5) | 9.2x10^-6^ | -0.7 ± 0.2 | 4.2x10^-6^ | 6.6 ± 0.2 | -0.7 ± 0.1 | 2.7x10^-6^ |
| CCI-onsite^1^ | 105 | 6.4 ± 0.2 | -0.7 (1.4) | 4.9x10^-7^ | -0.7 ± 0.2 | 1.1x10^-5^ | 6.6 ± 0.2 | -0.6 ± 0.1 | 3.0x10^-6^ |
| Usual care^1^ | 72 | 8.3 ± 0.3 | -0.1 (1.6) | 0.76 | -0.1 ± 0.2 | 0.74 | 8.1 ± 0.3 | -0.1 ± 0.2 | 0.76 |
| CCI-web vs. CCI-onsite^2^ |  |  | 0 ± 0.2 | 0.92 | 0 ± 0.2 | 0.90 |  | 0 ± 0.2 | 0.84 |
| CCI-web vs. usual care^2^ |  |  | -0.6 ± 0.2 | 0.01 | -0.6 ± 0.2 | 0.008 |  | -0.6 ± 0.2 | 0.006 |
| CCI-onsite vs. usual care^2^ |  |  | -0.6 ± 0.2 | 0.006 | -0.6 ± 0.2 | 0.01 |  | -0.6 ± 0.2 | 0.008 |
| CCI-all vs. usual care^2^ |  |  | -0.6 ± 0.2 | 0.003 | -0.6 ± 0.2 | 0.004 |  | -0.6 ± 0.2 | 0.003 |
|  |  |  |  |  |  |  |  |  |  |
| **∆10-year ASCVD risk** |  |  |  |  |  |  |  |  |  |
| CCI-all education^1^ | 135 | 10.5 ± 0.7 | -1.6 (5.4) | 0.0004 | -1.5 ± 0.6 | 0.01 | 9.6 ± 0.5 | -1.3 ± 0.3 | 4.9x10^-5^ |
| CCI-web^1^ | 53 | 10.7 ± 1.0 | -0.8 (4.6) | 0.24 | -0.6 ± 0.9 | 0.50 | 9.3 ± 0.7 | -1.1 ± 0.4 | 0.01 |
| CCI-onsite^1^ | 82 | 10.3 ± 0.8 | -2.2 (5.8) | 0.0005 | -2.2 ± 0.8 | 0.006 | 9.8 ± 0.7 | -1.5 ± 0.5 | 0.001 |
| Usual care^1^ | 55 | 12.7 ± 1.5 | 1.4 (9.3) | 0.28 | 1.1 ± 1.0 | 0.27 | 12.9 ± 1.2 | 1.2 ± 0.9 | 0.17 |
| CCI-web vs. CCI-onsite^2^ |  |  | 1.5 ± 0.9 | 0.1 | 1.5 ± 1.2 | 0.2 |  | 0.5 ± 0.6 | 0.45 |
| CCI-web vs. usual care^2^ |  |  | -2.1 ± 1.4 | 0.14 | -1.7 ± 1.4 | 0.21 |  | -2.3 ± 1.0 | 0.02 |
| CCI-onsite vs. usual care^2^ |  |  | -3.6 ± 1.4 | 0.01 | -3.3 ± 1.3 | 0.01 |  | -2.8 ± 1.0 | 0.007 |
| CCI-all vs. usual care^2^ |  |  | -3.0 ± 1.3 | 0.03 | -2.6 ± 1.2 | 0.03 |  | -2.6 ± 1.0 | 0.008 |
|  |  |  |  |  |  |  |  |  |  |
| **∆CIMT-average (mm)** |  |  |  |  |  |  |  |  |  |
| CCI-all education^1^ | 144 | 0.695 ± 0.009 | 0.002 (0.055) | 0.63 | 0.003 ± 0.004 | 0.45 | 0.685 ± 0.01 | 0.002 ± 0.004 | 0.65 |
| CCI-web^1^ | 65 | 0.683 ± 0.012 | 0.000 (0.056) | 0.95 | 0.003 ± 0.006 | 0.62 | 0.677 ± 0.014 | 0.003 ± 0.007 | 0.66 |
| CCI-onsite^1^ | 79 | 0.704 ± 0.014 | 0.004 (0.055) | 0.55 | 0.003 ± 0.006 | 0.62 | 0.692 ± 0.014 | 0.001 ± 0.006 | 0.84 |
| Usual care^1^ | 68 | 0.680 ± 0.013 | 0.004 (0.041) | 0.37 | 0.002 ± 0.006 | 0.74 | 0.68 ± 0.013 | 0.001 ± 0.006 | 0.87 |
| CCI-web vs. CCI-onsite^2^ |  |  | -0.003 ± 0.009 | 0.72 | 0.000 ± 0.009 | 0.97 |  | 0.002 ± 0.009 | 0.84 |
| CCI-web vs. usual care^2^ |  |  | -0.004 ± 0.009 | 0.64 | 0.001 ± 0.009 | 0.9 |  | 0.000 ± 0.009 | 0.82 |
| CCI-onsite vs. usual care^2^ |  |  | -0.001 ± 0.008 | 0.92 | 0.001 ± 0.009 | 0.87 |  | 0.000 ± 0.008 | 0.98 |
| CCI-all vs. usual care^2^ |  |  | -0.002 ± 0.007 | 0.74 | 0.001 ± 0.008 | 0.87 |  | 0.001 ± 0.007 | 0.88 |
|  |  |  |  |  |  |  |  |  |  |
| **∆Statin (%)** |  |  |  |  |  |  |  |  |  |
| CCI-all education^1^ | 218 | 48.2 ± 3.4 | -3.7 (34.4) | 0.12 | -3.6 ± 2.4 | 0.13 | 46.7 ± 3.2 | -3.3 ± 2.3 | 0.15 |
| CCI-web^1^ | 104 | 47.1 ± 4.9 | -3.9 (36.7) | 0.28 | -3.7 ± 3.5 | 0.30 | 44.8 ± 4.7 | -2.8 ± 3.5 | 0.43 |
| CCI-onsite^1^ | 114 | 49.1 ± 4.7 | -3.5 (32.4) | 0.25 | -3.6 ± 3.4 | 0.28 | 48.4 ± 4.5 | -3.8 ± 3.1 | 0.22 |
| Usual care^1^ | 73 | 64.4 ± 5.6 | 9.6 (37.9) | 0.03 | 9.5 ± 4.3 | 0.03 | 67.4 ± 5.4 | 8.8 ± 4.3 | 0.04 |
| CCI-web vs. CCI-onsite^2^ |  |  | -0.3 ± 4.7 | 0.94 | 0 ± 4.9 | 0.99 |  | 1.0 ± 4.7 | 0.83 |
| CCI-web vs. usual care^2^ |  |  | -13.4 ± 5.7 | 0.02 | -13.2 ± 5.6 | 0.02 |  | -11.6 ± 5.6 | 0.04 |
| CCI-onsite vs. usual care^2^ |  |  | -13.1 ± 5.4 | 0.01 | -13.2 ± 5.6 | 0.02 |  | -12.6 ± 5.3 | 0.02 |
| CCI-all vs. usual care^2^ |  |  | -13.3 ± 5.0 | 0.008 | -13.2 ± 5.0 | 0.009 |  | -12.1 ± 4.9 | 0.01 |
|  |  |  |  |  |  |  |  |  |  |
| **∆ Any antihypertensive medication (%)** |  |  |  |  |  |  |  |  |  |
| CCI-all education^1^ | 218 | 56.4 ± 3.4 | -11.9 (42.3) | 3.2x10^-5^ | -11.9 ± 2.9 | 3.6x10^-5^ | 55.8 ± 3.3 | -11.4 ± 2.8 | 5.3x10^-5^ |
| CCI-web^1^ | 104 | 55.8 ± 4.9 | -9.6 (40.7) | 0.02 | -9.9 ± 4.2 | 0.02 | 55.2 ± 4.8 | -9.8 ± 4.0 | 0.01 |
| CCI-onsite^1^ | 114 | 57.0 ± 4.7 | -14.0 (43.9) | 0.0006 | -13.9 ± 4.0 | 0.0005 | 56.2 ± 4.6 | -12.9 ± 4.0 | 0.001 |
| Usual care^1^ | 73 | 60.3 ± 5.8 | 9.6 (41.4) | 0.05 | 9.6 ± 5.1 | 0.06 | 61.2 ± 5.6 | 8.3 ± 4.8 | 0.09 |
| CCI-web vs. CCI-onsite^2^ |  |  | 4.4 ± 5.7 | 0.44 | 4.0 ± 5.8 | 0.49 |  | 3.1 ± 5.7 | 0.59 |
| CCI-web vs. usual care^2^ |  |  | -19.2 ± 6.3 | 0.002 | -19.5 ± 6.7 | 0.004 |  | -18.2 ± 6.3 | 0.004 |
| CCI-onsite vs. usual care^2^ |  |  | -23.6 ± 6.4 | 0.0002 | -23.6 ± 6.6 | 0.0005 |  | -21.2 ± 6.3 | 0.0008 |
| CCI-all vs. usual care^2^ |  |  | -21.5 ± 5.6 | 0.0002 | -21.6 ± 6.0 | 0.0004 |  | -19.7 ± 5.6 | 0.0004 |
|  |  |  |  |  |  |  |  |  |  |
| **∆ACE or ARB (%)** |  |  |  |  |  |  |  |  |  |
| CCI-all education^1^ | 218 | 28.9 ± 3.1 | 0.9 (27.1) | 0.62 | 1.5 ± 1.9 | 0.42 | 30.0 ± 2.9 | 0.6 ± 1.9 | 0.76 |
| CCI-web^1^ | 104 | 29.8 ± 4.5 | -1.0 (26.1) | 0.71 | -0.5 ± 2.7 | 0.86 | 31.3 ± 4.3 | -1.3 ± 2.6 | 0.63 |
| CCI-onsite^1^ | 114 | 28.1 ± 4.2 | 2.6 (28.1) | 0.32 | 3.4 ± 2.6 | 0.19 | 28.7 ± 4.0 | 2.3 ± 2.6 | 0.39 |
| Usual care^1^ | 73 | 21.9 ± 4.9 | 5.5 (28.3) | 0.1 | 3.7 ± 3.3 | 0.27 | 23.4 ± 4.7 | 5.0 ± 3.3 | 0.13 |
| CCI-web vs. CCI-onsite^2^ |  |  | -3.6 ± 3.7 | 0.33 | -3.9 ± 3.8 | 0.30 |  | -3.5 ± 3.7 | 0.34 |
| CCI-web vs. usual care^2^ |  |  | -6.4 ± 4.2 | 0.12 | -4.1 ± 4.3 | 0.35 |  | -6.3 ± 4.2 | 0.14 |
| CCI-onsite vs. usual care^2^ |  |  | -2.9 ± 4.2 | 0.50 | -0.2 ± 4.3 | 0.96 |  | -2.7 ± 4.2 | 0.52 |
| CCI-all vs. usual care^2^ |  |  | -4.6 ± 3.8 | 0.23 | -2.1 ± 3.9 | 0.59 |  | -4.4 ± 3.8 | 0.24 |
|  |  |  |  |  |  |  |  |  |  |
| **∆Diuretics (%)** |  |  |  |  |  |  |  |  |  |
| CCI-all education^1^ | 218 | 31.7 ± 3.2 | -9.6 (41.3) | 0.0006 | -9.5 ± 2.7 | 0.0004 | 31.2 ± 3.1 | -9.7 ± 2.7 | 0.0004 |
| CCI-web^1^ | 104 | 30.8 ± 4.6 | -10.6 (39.2) | 0.006 | -10.8 ± 3.9 | 0.005 | 29.9 ± 4.4 | -10.6 ± 3.8 | 0.005 |
| CCI-onsite^1^ | 114 | 32.5 ± 4.4 | -8.8 (43.2) | 0.04 | -8.3 ± 3.7 | 0.03 | 32.4 ± 4.3 | -8.8 ± 3.9 | 0.02 |
| Usual care^1^ | 73 | 30.1 ± 5.4 | 5.5 (32.9) | 0.16 | 5.2 ± 4.8 | 0.28 | 33.0 ± 5.3 | 3.2 ± 4.1 | 0.44 |
| CCI-web vs. CCI-onsite^2^ |  |  | -1.8 ± 5.6 | 0.75 | -2.5 ± 5.4 | 0.64 |  | -1.8 ± 5.4 | 0.74 |
| CCI-web vs. usual care^2^ |  |  | -16.1 ± 5.4 | 0.003 | -16.0 ± 6.2 | 0.01 |  | -13.8 ± 5.6 | 0.01 |
| CCI-onsite vs. usual care^2^ |  |  | -14.3 ± 5.6 | 0.01 | -13.4 ± 6.2 | 0.03 |  | -12.0 ± 5.7 | 0.03 |
| CCI-all vs. usual care^2^ |  |  | -15.1 ± 4.8 | 0.001 | -14.7 ± 5.6 | 0.009 |  | -12.8 ± 4.9 | 0.009 |
| ^1^ Means (standard deviations) or ± one standard error are presented. Sample sizes, means, and significance levels refer to subjects with baseline and one-year measurements for *completers*, and to 349 subjects (262 intervention and 87 usual care) for *all starters*. Significance levels for *completers* refer to one-sample t-test with or without adjustment. Untransformed triglyceride and C-reactive protein values are presented, however, their statistical significances were based on their log-transformed values. | | | | | | | | | |
| ^2^ Mean differences ± one standard error are presented. Significance levels refer to two-sample t-test or analysis of covariance for the differences. | | | | | | | | | |
| ^3^ Adjusted for sex, age, baseline BMI, baseline insulin use (user vs. non-user), and African-American race. | | | | | | | | | |
| ^4^ Imputed values based on 700 iterations from multivariate normal regression. | | | | | | | | | |
| ^5^ A significance level of P<0.0019 ensures overall simultaneous significance of P<0.05 over the 26 variables using Bonferroni correction. | | | | | | | | | |
