# Supplementary material for: Cardiovascular disease risk factor responses to a type 2 diabetes care model including nutritional ketosis induced by sustained carbohydrate restriction at 1 year: an open label, non-randomized, controlled study
Source: Cardiovasc Diabetol. 2018 May 1;17:56. doi: 10.1186/s12933-018-0698-8 (PMC5928595; doi:10.1186/s12933-018-0698-8)
Supplement: Supplementary file 3 — Additional file 3: Figure S1. Distribution of changes in selected biomarkers for CCI and UC completers. Histograms of changes at one year for CCI (blue) and UC (gray) are overlaid. Very few (≤1%) CCI participants demonstrated changes in an undesirable direction at one year that were outside the range of changes observed in the UC group for key lipid and lipoprotein particles. (A) Apolipoprotein B (B) Apolipoprotein A1 (C) Apolipoprotein B/Apolipoprotein A1 ratio (D) LDL-P (E) Small LDL-P (F) Large VLDL-P (G) Triglyceride/HDL-C ratio. [file 12933_2018_698_MOESM3_ESM.pdf]

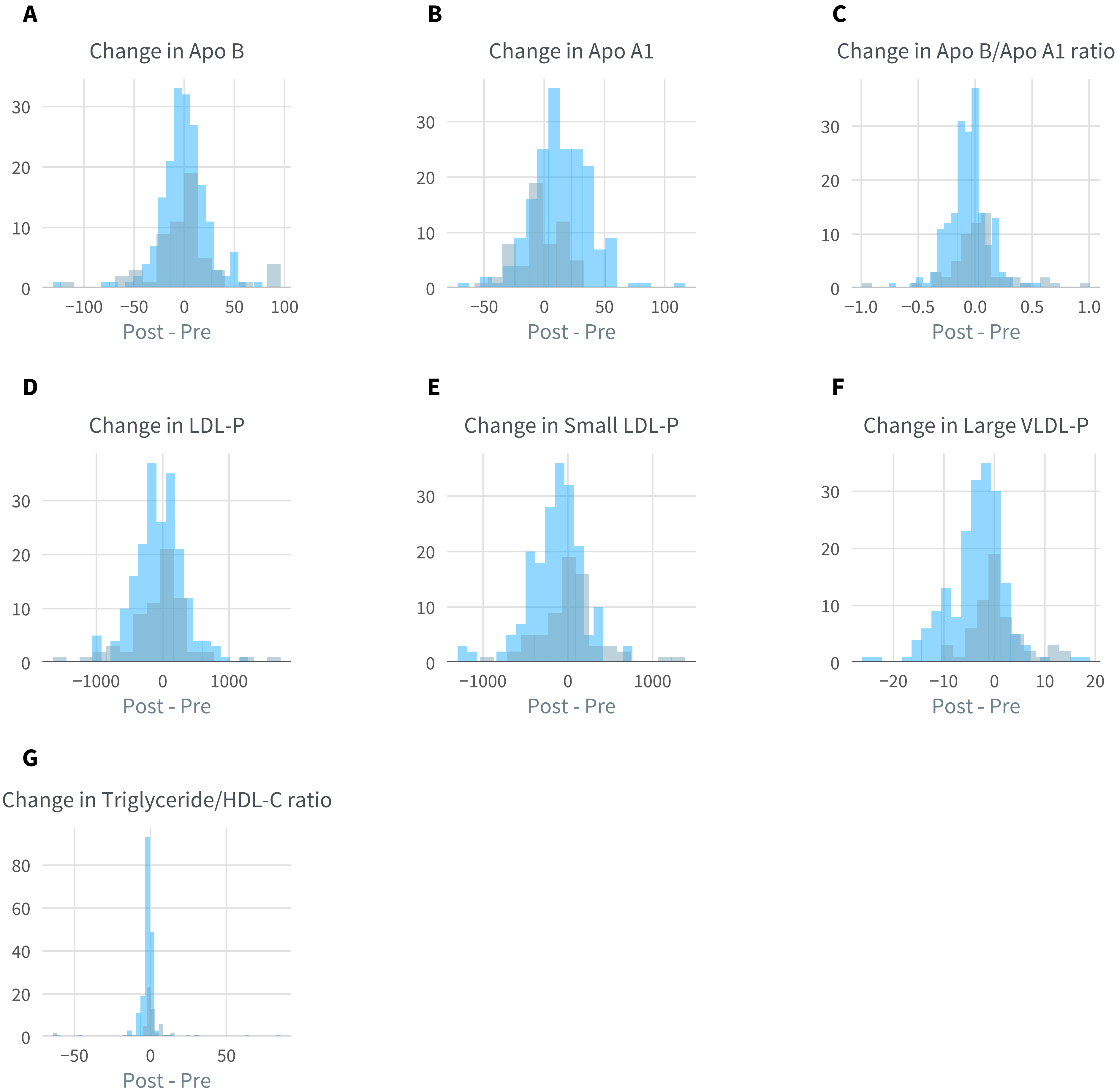
A
Change in Apo B
30
20
10
0
−100
−50
0
50
100
Post - Pre
B
Change in Apo A1
30
20
10
0
−50
0
50
100
Post - Pre
C
Change in Apo B/Apo A1 ratio
30
20
10
0
−1.0
−0.5
0.0
0.5
1.0
Post - Pre
D
Change in LDL-P
30
20
10
0
−1000
0
1000
Post - Pre
E
Change in Small LDL-P
30
20
10
0
−1000
0
1000
Post - Pre
F
Change in Large VLDL-P
30
20
10
0
−20
−10
0
10
20
Post - Pre
G
Change in Triglyceride/HDL-C ratio
80
60
40
20
0
−50
0
50
Post - Pre
